# A novel *KCNC3* gene variant in the voltage-dependent Kv3.3 channel in an atypical form of SCA13 with dominant central vertigo

**DOI:** 10.3389/fncel.2024.1441257

**Published:** 2024-10-02

**Authors:** Felix P. Bernhard, Sven Schütte, Moritz Heidenblut, Moritz Oehme, Susanne Rinné, Niels Decher

**Affiliations:** ^1^Department of Psychiatry and Psychotherapy, Philipps-University Marburg, Marburg, Germany; ^2^Institute for Physiology and Pathophysiology and Center for Mind Brain and Behavior (CMBB), Philipps-University Marburg, Marburg, Germany

**Keywords:** potassium channels, neurological disorders, *KCNC3*, Kv3.3, rotational vertigo

## Abstract

Potassium channel mutations play an important role in neurological diseases, such as spinocerebellar ataxia (SCA). SCA is a heterogeneous autosomal-dominant neurodegenerative disorder with multiple sub-entities, such as SCA13, which is characterized by mutations in the voltage-gated potassium channel Kv3.3 (*KCNC3*). In this study, we present a rare and atypical case of SCA13 with a predominant episodic central rotational vertigo, while the patient suffered only from mild progressive cerebellar symptoms, such as dysarthria, ataxia of gait and stand, and recently a cognitive impairment. In this patient, we identified a heterozygous variant in *KCNC3* (c.2023G > A, p.Glu675Lys) by next-generation sequencing. This Kv3.3^E675K^ variant was studied using voltage-clamp recordings in *Xenopus* oocytes. While typical SCA13 variants are dominant-negative, show shifts in the voltage-dependence of activation or an altered TBK1 regulation, the Kv3.3^E675K^ variant caused only a reduction in current amplitude and a more pronounced cumulative inactivation. Thus, the differences to phenotypes observed in patients with classical SCA13 mutations may be related to the mechanism of the observed Kv3.3 loss-of-function. Treatment of our patient with riluzole, a drug that is known to also activate potassium channels, turned out to be partly beneficial. Strikingly, we found that the Kv3.3 and Kv3.3^E675K^ inactivation and the frequency-dependent cumulative inactivation was antagonized by increased extracellular potassium levels. Thus, and most importantly, carefully elevated plasma potassium levels in the physiological range, or novel drugs attenuating Kv3.3 inactivation might provide novel therapeutic approaches to rescue potassium currents of SCA13 variants *per se*. In addition, our findings broaden the phenotypic spectrum of Kv3.3 variants, expanding it to atypical phenotypes of Kv3.3-associated neurological disorders.

## 1 Introduction

In the fields of neuroscience and clinical neurology, the intricate workings of ion channels (Graves and Hanna, 2005), particularly potassium channels, hold profound implications for human health and disease (Attali, 1996; Shieh et al., 2000). Recent research has focused on Kv channels, such as Kv1.2, in the context of *KCNA2* mutations. These mutations, depending on whether they result in gain- or loss-of-function, cause epilepsy, developmental disorders (Masnada et al., 2017), and neurodegenerative diseases such as spinocerebellar ataxia, underscoring their clinical relevance (Helbig et al., 2016). More recently, Kv3.3 channels have gained importance in clinical and pathogenetic investigations. Kv3.3 channels, encoded by the *KCNC3* gene, are critical in modulating neuronal membrane potential and regulating action potential firing, thus maintaining normal brain function. Investigations have revealed that Kv3.3 channels are crucial in the pathogenesis of developmental and neurodegenerative disorders, as described in an (French family with) adult onset-ataxia and cognitive delay and impairment (Herman-Bert et al., 2000) and in a Filipino family with cerebellar ataxia (Waters et al., 2005), highlighting the genetic mechanisms underlying these neurological disorders (Masnada et al., 2017). In common, all of these cases express brain stem and cerebellar dysfunction and have been historically early categorized within autosomal dominant cerebellar ataxia (ADCA) (Harding, 1993).

Spinocerebellar Ataxia (SCA) have been defined later, dependent on genetic transmission and can manifest through various inheritance patterns including autosomal dominant (AD), autosomal recessive (AR), X-linked, or mitochondrial modes. As a result, SCA is observed within a heterogeneous group of ataxia. In addition to a wide variety of specific genes that cause SCA, a common pathophysiological and neuronal correlate is the loss of Purkinje cells, leading toward the clinical manifestation of cerebellar dysfunction: ataxia of gait, balance, coordination of limb movements, speech and coordination of the oculomotor system (Huang and Verbeek, 2019; Rudaks et al., 2024). Loss of Purkinje cells is often observed within MRI-Imaging due to cerebellar atrophy (Paulson et al., 2017). In addition to cerebellar symptoms, motor-neuronal degeneration and pyramidal signs, movement disorders such as parkinsonism, peripheral neuropathy or non-neurological symptoms can be present (Teive et al., 2019).

Spinocerebellar ataxia type 13 (SCA13) is an autosomal dominant neurodegenerative disorder characterized by cerebellar atrophy, particularly affecting the cerebellar vermis, resulting in a cerebellar syndrome with ataxia, dysarthria and nystagmus (Middlebrooks et al., 2013). Additional features may include pyramidal signs, epilepsy, auditory deficits, and mild intellectual disability. SCA13 results from mutations in the voltage-gated potassium channel *KCNC*3 (Kv3.3), with a prevalence of about 1.5% in Europe.

Kv3 subfamily members of voltage-gated potassium channels are typically expressed in neurons capable of high firing rates (Kaczmarek and Zhang, 2017). Kv3.3 is predominantly found in Purkinje cells in the cerebellum and in nuclei of the auditory brainstem. Kv3.3 channels primarily conduct outward potassium currents and contribute to the repolarization phase of action potentials. Their activity is critical for the regulation of neuronal excitability, particularly in inhibitory interneurons within the central nervous system. Kv channels can inactivate with a slow (C-type) or fast (N-type) inactivation. The N-type is achieved by the so-called “ball and chain” mechanism, in which an amino-terminal amino acid sequence blocks the channel pore upon depolarization (Sukomon et al., 2023). The mRNA of Kv3.3 contains two individual putative start codons for methionine (Rudy et al., 1999; Rae and Shepard, 2000) and it has been demonstrated, that alternative translation initiation (ATI) from the two different start codons of Kv3.3 leads to differences in channel inactivation (Rudy et al., 1999; Fernandez et al., 2003) as N-type inactivation can only occur in the long channel variant, containing the inactivation ball.

Three distinct SCA13 phenotypes have been described. The first manifests in childhood with cerebellar hypoplasia, non-progressive limb ataxia, trunk ataxia, and gait disturbances, often accompanied by moderate intellectual disability. The second phenotype features slowly progressive cerebellar atrophy in childhood, with ataxia, dysarthria, delayed motor milestones, and mild to moderate intellectual disability. Finally, the third phenotype presents in adulthood with cerebellar atrophy, ataxia, and spasticity. The phenotype of SCA13, particularly in adulthood, may mimic the clinical presentation of hydropic inner ear disease in its early stages, presenting with nystagmus, auditory impairment, and vertigo symptoms.

Different disease-causing mutations in *KCNC3* have been identified and functionally studied: p.D129N (Duarri et al., 2015), p.R420H (Waters et al., 2006), p.R423H (Figueroa et al., 2010; Figueroa et al., 2011), p.T428I (Nemeth et al., 2013; Parolin Schnekenberg et al., 2015), p.F448L (Waters et al., 2006; Figueroa et al., 2010), p.V535M (Duarri et al., 2015), p.S591G (Duarri et al., 2015) and p.G592R (Zhang et al., 2016; Zhang et al., 2021b). Interestingly, these mutations in *KCNC3* show quite heterogeneous effects on the Kv3.3 channel function, causing both, gain- and loss-of-functions (Supplementary Table 1). The pathological consequences of these channel alterations have been validated by experimental studies in transgenic zebrafish and cellular models, revealing deficits in motor control due to suppressed excitability of motor neurons, defective axonal pathways, and reduced current amplitudes. In addition, studies in mice have demonstrated disrupted dendritic development and incipient cell death.

It has previously been reported, that Kv3.3 activates cerebellar TANK-binding kinase 1 (TBK1) and that this stimulation is largely increased in the disease-causing Kv3.3^G592R^ mutant (Zhang et al., 2016; Zhang et al., 2021b). TBK1 is a serine/threonine protein kinase that regulates several cellular processes and TBK1 dysfunction can trigger neurodegeneration (Li et al., 2016; Oakes et al., 2017). On the other hand, TBK1 activity is required for Kv3.3 to bind to its auxiliary subunit Hax-1, preventing channel inactivation. Hax-1 is an anti-apoptotic protein required for the survival of cerebellar neurons. Overactivation of TBK1 by mutant Kv3.3 channels leads to the loss of Hax-1 binding to the channel by its accumulation in multivesicular bodies and lysosomes and stimulates the production of exosomes and increases the rate of cell death (Zhang et al., 2021b).

In the current study we report a patient with hereditary atypical SCA13 and a predominant central vertigo. Here, genetic testing revealed a heterozygous variant in *KCNC3*, which encodes a voltage-gated potassium channel essential for neuronal excitability regulation. This finding suggests a potential role for Kv3.3 channel dysfunction in cases of atypical SCA13 and/or isolated vertigo, challenging conventional diagnostic paradigms and highlighting the complex genetic architecture underlying vestibular disturbances.

## 2 Materials and methods

### 2.1 Gene diagnostic and sequence analysis

We did not perform a clinical study involving human participants. The individual patient described in this case provided a written informed consent to participate in this scientific study.

Genetic diagnostics were performed in the centers for human genetics of SynLab in Mannheim and Bad Nauheim. Additional molecular repeat analyses for certain SCA genes were performed by the Department for Human Genetics at the University Clinic of Tübingen.

Clinical exome analysis was performed using next-generation sequencing (NGS) techniques. A target-specific gene library was created utilizing the “TruSight Nextera Flex for Enrichment Technology/TruSight One Kit” by Illumina. The sequencing process was carried out with an Illumina Sequencer (NextSeq 500). Genetic data analysis involved comparison of the sequences obtained with the reference genome NCBI37/hg19 using the Varvis 1.10.1 software. Copy number variation (CNV) analysis was performed using the CNV module within the Varvis software. The classification of genetic variations was conducted in accordance with the ACMG guidelines (Richards et al., 2015). Classification of variants was based on clinical and familial data of the patient, compared with existing scientific literature and international databases. Variants falling under class 1 and 2, as well as synonymous variations considered irrelevant for mRNA splicing based on *in silico* predictions and current scientific knowledge, were not reported.

### 2.2 Allele frequency, amino acid sequence alignment and pathogenicity prediction

Allele frequencies were obtained from the EVS server (Exome Variant Server)^1^ or the gnomAD database (gnomAD).^2^ Sequence alignment of human (NM_004977), *Mus musculus* (NM_008422.3) *Rattus norvegicus* (NM_053997.5), *Bos taurus* (XM_027515050.1), *Pan troglodytes* (XM_054672643.2), *Canis lupus familiaris* (XM_038656201.1) and *Danio rerio* (NP_001182170) Kv3.3 proteins was performed with Clustal Omega.^3^ The pathogenicity of the Kv3.3^E675K^, USH2A^V2306L^ and LOXHD1^A553V^ variants was predicted using SIFT, PolyPhen-2 and MutationTaster.

### 2.3 Cloning and site-directed mutagenesis

Human (h) Kv3.3 (NM_004977) was subcloned from the open reading frame (ORF) cDNA clone (Origene, RG222238) into the oocyte expression vector pSGEM using EcoRI and XhoI restriction enzymes (Fermentas). Mutations were introduced using the QuikChange Site-Directed Mutagenesis Kit (Agilent) and confirmed by Sanger sequencing (Seqlab).

### 2.4 Isolation of *Xenopus laevis* oocytes

The animal study with *Xenopus laevis* toads was approved by the Ethics Committee of the Regierungspräsidium Gießen (protocol code V54-19c 20 15 h 02 MR 20/28 Nr.A 23/2017, approved on 12.02.2018). Oocytes were isolated from anesthetized *Xenopus laevis* frogs and then incubated in OR2 solution containing in mM: 82.5 NaCl, 2 KCl, 1 MgCl_2_, 5 HEPES; pH 7.5 with NaOH, supplemented with collagenase (1.5 mg/mL) (Nordmark) to remove residual connective tissue. The oocytes were then stored in ND96 solution containing in mM: 96 NaCl, 2 KCl, 1.8 CaCl_2_, 1 MgCl_2_, 5 HEPES; pH 7.4 with NaOH, supplemented with Na-pyruvate (275 mg/L), theophylline (90 mg/L) and gentamicin (50 mg/L) at 18°C.

### 2.5 cRNA synthesis and injection

Kv3.3 wild-type or mutant cDNAs were linearized with NheI and cRNA was synthesized using the HiScribe T7 ARCA mRNA Kit (New England Biolabs). Quantity and quality of cRNAs were assessed by agarose gel electrophoresis and spectroscopy (NanoDrop 2000c, Thermo Fisher Scientific). Stage IV and V oocytes were each injected with 50 nL of cRNA each using a Nanoject II microinjector (Süd-Laborbedarf GmbH).

### 2.6 Two-electrode voltage-clamp recordings in *Xenopus laevis* oocytes

All voltage-clamp recordings were performed at room temperature (20–22°C) using an Axon Axoclamp 900A Microelectrode Amplifier (Molecular Devices) and a Digidata 1440 Series (Axon Instruments) as an analog/digital converter or with a TurboTEC 10CD (npi) amplifier and a Digidata 1200 Series (Axon Instruments). Micropipettes were made from borosilicate glass capillaries (GB 150TF-8P, Science Products) and pulled using a DMZ-Universal Puller (Zeitz). The recording pipettes had a resistance of 0.5–1.0 MΩ when filled with 3 M KCl solution. ND96 (pH 7.5) was used as recording solution. Data were acquired with Clampex 10 (Molecular Devices) and analyzed with Clampfit 10 (Molecular Devices) and Origin 2016 (OriginLab Corp.). To analyze the kinetics of the recovery from inactivation the voltage was stepped from a holding potential of −80 mV to +50 mV for 200 ms, followed by another step from −80 mV to +50 mV after 20 ms. The depolarization step was repeated 17 times with increasing time for recovery, with an increment of 500 ms per step (Supplementary Figure 5F). The inter-sweep time interval was 30 s to guarantee complete inactivation recovery from the second pulse. For each sweep, the relative recovered current *I*_rec_ was calculated by building the ratio of the peak current amplitude of the first *I*_1max_ and the second depolarizing voltage step *I*_2max_ after subtraction with the minimal current amplitude *I*_1min_ at the end of the first voltage step (Equation 1):


(1) $$I_{rec}=\left(I_{1\max}-I_{1\min}\right)/\left(I_{2\max}-I_{1\min}\right)$$

A mono-exponential fit was used to analyze the time constants of current recovery. To record the current–voltage relationship (IV) the voltage was stepped for 500 ms from a holding potential of −80 mV to −60 mv to +80 mV in 20 mV increments (Supplementary Figure 5B). To analyze the conductance-voltage relationship (GV) the voltage was stepped for 25 ms from a holding potential of −80 mV to −70 to +60 mV, in 10 mV increments. The depolarization step was followed by a voltage step to −40 mV (or alternatively −25 or −15 mV for tail currents of small amplitude) for 400 ms to record the tail currents (Supplementary Figure 5A). Peak tail current amplitudes were analyzed for all applied voltages and normalized to the maximal current amplitude. The voltage at half-maximal activation (V_1/2_) and the slope of the Boltzmann fit (*k* value) was calculated using the Boltzmann equation. Both, the IV and the GV protocol, had an inter-sweep time interval of 7 s.

To analyze the voltage-dependence of inactivation, the voltage was stepped for 1 s from a holding potential of −80 mV to −100 to +30 mV with 10 mV increments [for the Kv3.3^M1I^ variant 10 s steps were applied, as this variant does not show fast inactivation (Supplementary Figure 5D)]. The inter-sweep time interval was set to 10 s. Subsequently the voltage was stepped to +50 mV for 400 ms (Supplementary Figure 5C). Peak current amplitudes were analyzed at the second step, respectively and normalized to the maximal current amplitude. The voltage at half maximal inactivation (V_1/2_) and the slope of the Boltzmann fit (*k*) were calculated using the Boltzmann equation.

To quantify current amplitudes voltage was stepped for 200 ms from a holding potential of −80 mV to +50 mV (Supplementary Figure 5E). The peak current amplitude was analyzed and normalized to the mean current amplitude of Kv3.3 wild-type or Kv3.3^M1I^ or Kv3.3^M77I^ as indicated for each batch.

The TBK1 inhibitor MRT67307 (Millipore) was dissolved in DMSO. 24 h after cRNA injection, oocytes were incubated in ND96 solution containing 40 μM MRT67307 for 30 min and then recorded using the IV protocol. The current amplitude was normalized to the peak current amplitude. A bi-exponential fit was used to analyze the time constants.

Pharmacological modulation of Kv3.3 and the E675K variant were measured using the protocols mentioned above. Niflumic acid (100 mM stock, Sigma), carbamazepine (100 mM stock, Thermo Scientific) and riluzole (100 mM stock, TCI chemicals) were dissolved in DMSO. 4-aminopyridine (4-AP, Sigma) was dissolved in ddH_2_O (1 M stock). Prior to the measurements, the stocks were diluted in ND96 solution according to the desired final concentrations.

Extracellular potassium sensitivity of wild-type Kv3.3 and Kv3.3^E675K^ was measured using modified ND96 solutions with varying potassium concentrations (1 mM, 2 mM, 4 mM, 8 mM, 98 mM) by replacing NaCl with KCl. To account for the consequential shift in reversal potential and the accompanying changes in driving force, the GV protocol was slightly adjusted for the different potassium concentrations by changing the voltage step for the tail current analysis (1 mM K^+^ = −25 mV, 8 mM K^+^ = −15 mV, 98 mM K^+^ = −40 mV). Extracellular potassium dependent changes in inactivation kinetics and recovery from inactivation were recorded and analyzed as described above. The time constant *τ* of the inactivation recovery was calculated using a mono-exponential fit in Origin 2016. To analyze the frequency dependence of inactivation accumulation, voltage was stepped for 5 ms from a holding potential of −80 mV to +40 mV with varying inter-sweep time intervals according to the required frequency (1 Hz = 1000 ms, 10 Hz = 100 ms, 50 Hz = 20 ms, 100 Hz = 10 ms) (Supplementary Figure 5G). Current amplitudes were analyzed at the end of the voltage step and normalized to the maximal current amplitude. Assuming an independence from extracellular potassium concentration, the theoretical reduction in current by changes in the driving force can be calculated based on the Goldman–Hodgkin–Katz equation as it was done by Larsen and colleagues (Larsen et al., 2011) (Equation 2):


(2) $$\frac{I_{C1}}{I_{C2}}=\frac{{\left[K^{+}\right]}_{in}^{C1}-{\left[K^{+}\right]}_{ex}^{C1}*\exp\left(\frac{-V_{m}*F}{R*T}\right)}{{\left[K^{+}\right]}_{in}^{C2}-{\left[K^{+}\right]}_{ex}^{C2}*\exp\left(\frac{-V_{m}*F}{R*T}\right)}$$

The equation is used to calculate the theoretical current ratio $\frac{I_{C1}}{I_{C2}}$ of two different extracellular potassium concentrations ${\left[K^{+}\right]}_{ex}^{C1}$ and ${\left[K^{+}\right]}_{ex}^{C2}$ using an intracellular potassium concentration ${\left[K^{+}\right]}_{in}$ of 108.6 mM, based on the concentrations in *Xenopus laevis* oocytes gathered by Weber (1999). $V_{m}$ is the utilized membrane potential, F the faraday constant, R the gas constant, and T the temperature. The time constant *τ* of the first 2 s of cumulative inactivation was calculated using a mono-exponential fit in Origin 2016 software.

### 2.7 Statistical analyses

Values are expressed as mean ± s.e.m. For oocyte experiments N ≥ 3 different batches were used. Sample size was not predetermined, and the number of experiments required was estimated based on previous experiments and literature in this field. There were no exclusion criteria and no data were excluded from the analysis. No randomization or blinding was applied to the experiments. Statistical analysis was performed using Microsoft Excel 2013 and OriginPro 2016. The respective graphs show the number of replicates (*n*) from different samples. Significances are indicated by asterisks, which indicate the level of significance as follows: n.s., not significant, *p < 0.05; **p < 0.01; ***p < 0.001.

## 3 Results

### 3.1 Clinical findings

The 55 years old patient’s medical journey commenced at the age of 27 with a gradual onset of symptoms, including progressive right sided hearing loss and rotational vertigo. Over the ensuing years, this evolved into severe right sided hearing loss, episodic rotational vertigo, and mild cerebellar ataxia. Notably, the rotational vertigo episodes escalated in both frequency (from once to twice daily) and intensity, lasting 30 min until hours. Initially misdiagnosed with Meniere’s disease, the individual underwent unsuccessful ototoxic and surgical interventions, including gentamicin treatment to both inner ears, saccotomies of the right vestibular organ in 1999 and 2003, saccotomies of the left vestibular organ in 2002 and 2004, and bilateral neurotomies of vestibulocochlear nerves in 2007 and 2009, leading to an iatrogenic injury of the left facial nerve.

Alongside these challenges, cerebellar symptoms such as ataxia of gait and stand have been additionally developed and mildly progressed. Given the clinical history and evolving doubts regarding the diagnosis of bilateral Meniere’s disease and as suspicions of an ataxia subtype had been raised, a genetic testing was initiated.

### 3.2 Actual clinical manifestation and diagnostic findings

**Figure 1:**
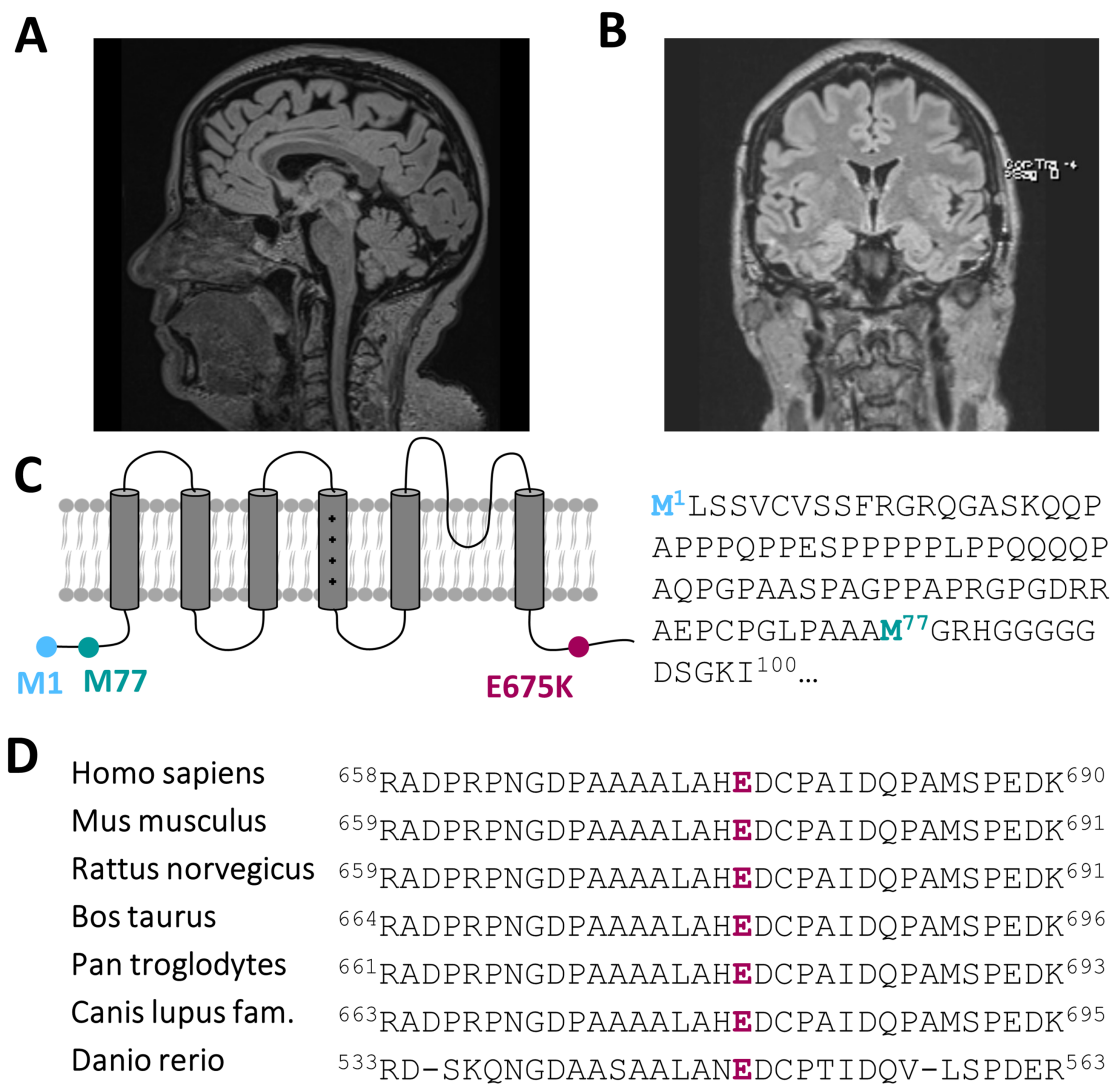
A heterozygous *KCNC3* variant in an atypical case of SCA13 with a dominant central rotational vertigo, only mild progressive cerebellar symptoms and an early onset of cognitive impairment. **(A)** Mid-sagittal T1/T2 and Flair-sequence MR image of the patient in 2024 showing a mild cerebellar atrophy and **(B)** a global reduction of brain volume. **(C)** Topology of a Kv3.3 channel subunit. The location of the E675K variant is highlighted in purple and the location of the alternative start of the N-terminus is highlighted in turquoise. **(D)** Partial amino acid sequence alignment of different Kv3.3 orthologues. The conserved E675 residue is highlighted in purple.

Recent developments within the last 1–2 years have seen the emergence of mild to moderate deficits in short-term memory and signs of amnesic aphasia. This could be investigated and verified by neuropsychological testing showing signs of beginning dementia of unclear underlying etiology. Dementia screening has been investigated, showing only a mild cerebellar atrophy (Figure 1A; Supplementary Figures 1A–D) and morphologic changes due to global loss of brain volume via MRI (Figure 1B), albeit without a prove of ß-amyloid-or tau pathology within cerebrospinal fluid analysis.

Further actual clinical examinations revealed truncal and extremity ataxia, alongside an unsteady gait and mild cerebellar dysarthria. No spontaneous nystagmus was observed, and a skew deviation was apparent during specific eye movements. The head impulse test was inconclusive due to a head tremor, while assessments for positional vertigo yielded unremarkable results. A skew deviation was observed upon upward and leftward gaze. Additionally, partial right facial nerve palsy was noted, reportedly unchanged since 2009. Herein, skew deviation and disconjugated eye movements were observed 2009, prompting further investigations via magnetic resonance imaging (MRI) of the cervical spine and brain with Acoustic Neuroma Protocol. However, no pathological findings were identified.

Audiometric assessments confirmed severe progressive hearing loss on the right side and indicated slight left sided impairment: Audiometric evaluation demonstrated left sided surditas and profound pan-tonal hearing loss in the right ear, approximately 70 dB. Compared to prior records, progressive bilateral hearing loss was documented. Given discrepancies between subjective and objective findings, Brainstem Auditory Evoked Response (BAER) testing was conducted in 2018. Results revealed a slight exacerbation of hearing loss in the right ear as previously noted, yet surditas in the left ear was not confirmed. Thus, progressive hearing loss exceeding 40 dB over several years was solely confirmed in the right ear. Caloric testing demonstrated reduced excitability on the left side.

### 3.3 Genetic findings

The index patient has two children: a son and a younger daughter. The son of the index patient (28 years old at the time of genetic diagnosis) carries at least the same genotype of the *KCNC3* variant (c.2023G > A, p.Glu675Lys). There are no symptoms identified in this variant carrier, which manifested in the index patient in the 2nd to 3rd decade of life. In addition, it might be that the variant has only a partial or sex-specific penetrance of the phenotype. No other family members are known to have the same symptoms of hearing impairment, ataxia or vertigo. gDNA of the parents of the index patient was not achievable and therefore investigations like trio-WES analyses were not realizable. To analyze the genetic origin of this disease, we performed a clinical exome analysis by next-generation sequencing (NGS). First, genes were categorized according to Human Phenotype Ontology (HPO) terms using search terms that describe the phenotype of the patient, like “ataxia,” “hearing impairment,” and “vertigo” to provide a clinical selection of the exome-analysis. Utilizing this approach and due to the atypical phenotype of our patient, the software analyzed a very large variety of 1098 syndromes with a genetic basis and screened for variants in 1114 genes. The genes that were screened by NGS and analyzed for variants are listed in Supplementary Table 2. The copy number variation (CNV) analysis for the studied genes did not show any signs of deletions or duplications.

The subsequent classification of variants was based on clinical and familial data of the patient, compared with existing scientific literature and public databases. We have identified three class 3 variances of unknown significance (VUS) in the genes *USH2A, LOXHD1* or *KCNC3*, which were all present in a heterozygous state. We identified a variant in the usherin gene (*USH2A*) with an *c.6916G > C* exchange, leading to a p.Val2306Leu substitution of a conserved amino acid within the fibronectin type III domain with a chemically similar amino acid. This variant was not documented in ClinVar and the literature. The variant was very rare in the gnomAD database, with an allele frequency of 6.2 × 10^−7^. Following *in silico* prediction analysis using SIFT, PolyPhen-2 and MutationTaster, the potential pathogenicity of this variant remains uncertain. Most importantly, the clinical features, in particular the central vertigo, are not in agreement with an Usher syndrome. In addition, mutations in the *USH2A* gene are causing Usher syndrome type 2 only in the homozygous state. Therefore, this exchange was not further considered as a disease-causative variant.

Another variant was found in the lipoxygenase homology PLAT domains 1 gene (*LOXHD1*) with a heterozygous *c.1658_1659delCCinsTT* change in the PLAT/LH2 domain, resulting in a physicochemical relative neutral p.Ala553Val exchange of a conserved amino acid. This variant was absent in ClinVar and the literature. The variant was very rare in the gnomAD database, with an allele frequency of 1.16 × 10^−5^. However, two of the *in silico* prediction tools that we used predicted a benign impact of this exchange. The clinical features, in particular the central vertigo, are not in agreement with a *LOXHD1* mutation. In light of the absence of a pathogenic *in silico* prediction of the variant and the fact that disease-causing *LOXHD1* mutations follow a recessive inheritance trait, this nucleotide exchange was also not further considered as a disease-causative variant.

Most importantly, we identified a variant in the SCA13-associated gene *KCNC3*, encoding the voltage-gated Kv3.3 channel, with a *c.2023G > A* nucleotide exchange, that causes a charge reversing p.Glu675Lys amino acid exchange of a highly conserved amino acid in the cytosolic C-terminus of the channel protein (Figures 1C,D). While this variant has not been reported in the literature, it is listed in ClinVar and classified with uncertain significance. The variant was very rare in the gnomAD database with an allele frequency of 3.34 × 10^−5^. In addition to its rare prevalence, two of the three *in silico* pathogenicity prediction tools we used predicted that variant to be probably damaging or as not tolerated. The lack of this variant in public databases, the physiochemical nature of the amino acid exchange and the partly pathogenic disease causing *in silico* predictions, together with the fact that SCA13 mutations are primarily found in the heterozygous state, indicate that the *KCNC3* variant is the most likely disease-associated variant in our patient with an atypical cerebellar ataxia.

Although mutations in *USH2A* and *LOXHD1* can be associated with mechanotransduction defects in cochlear hair cells and hearing loss (Mori et al., 2015; Hartel et al., 2016; Lee et al., 2020; Trouillet et al., 2021; Crane et al., 2023), any potential peripheral contributions to vertigo of the patient caused by mutations in these two genes can be excluded due to bilateral vestibular neurectomy, which supports the hypothesis of a central vertigo mediated by the *KCNC3* variant.

### 3.4 Early therapy approaches

Since the exact dysfunction due to the mutation was not yet described in the literature, treatment commenced with 4-aminopyridine (4-AP), a drug shown to be beneficial in patients with potassium channel gain-of-function mutations (Humphries and Dart, 2015). Despite initial treatment, no improvement was noted and symptoms increased dramatically: the patient was referred again to the hospital with severe rotational vertigo. Therefore, subsequent titration of carbamazepine was realized, in order to explore another pharmacological treatment toward a putative potassium-channel loss-of-function. This resulted in prolonged intervals between symptom-episodes (from daily manifestation to weekly manifestation) and reduction of symptom severity. One year later, medication was switched from carbamazepine to riluzole (50 mg, twice daily), with again a positive effect on symptom severity and frequency of manifestation. Currently, the patient also receives 47.5 mg of metoprolol in the morning as anti-hypertensive treatment, which did not interfere with the vertigo phenotype.

To investigate whether the *KCNC3* variant that we identified in the course of the therapeutic approaches leads to functional changes that are potentially disease-causing, we next analyzed the electrophysiological changes caused by the Kv3.3^E675K^ variant, also aiming to identify a novel therapy approach.

### 3.5 Kv3.3^E675K^ did not change channel gating

**Figure 2:**
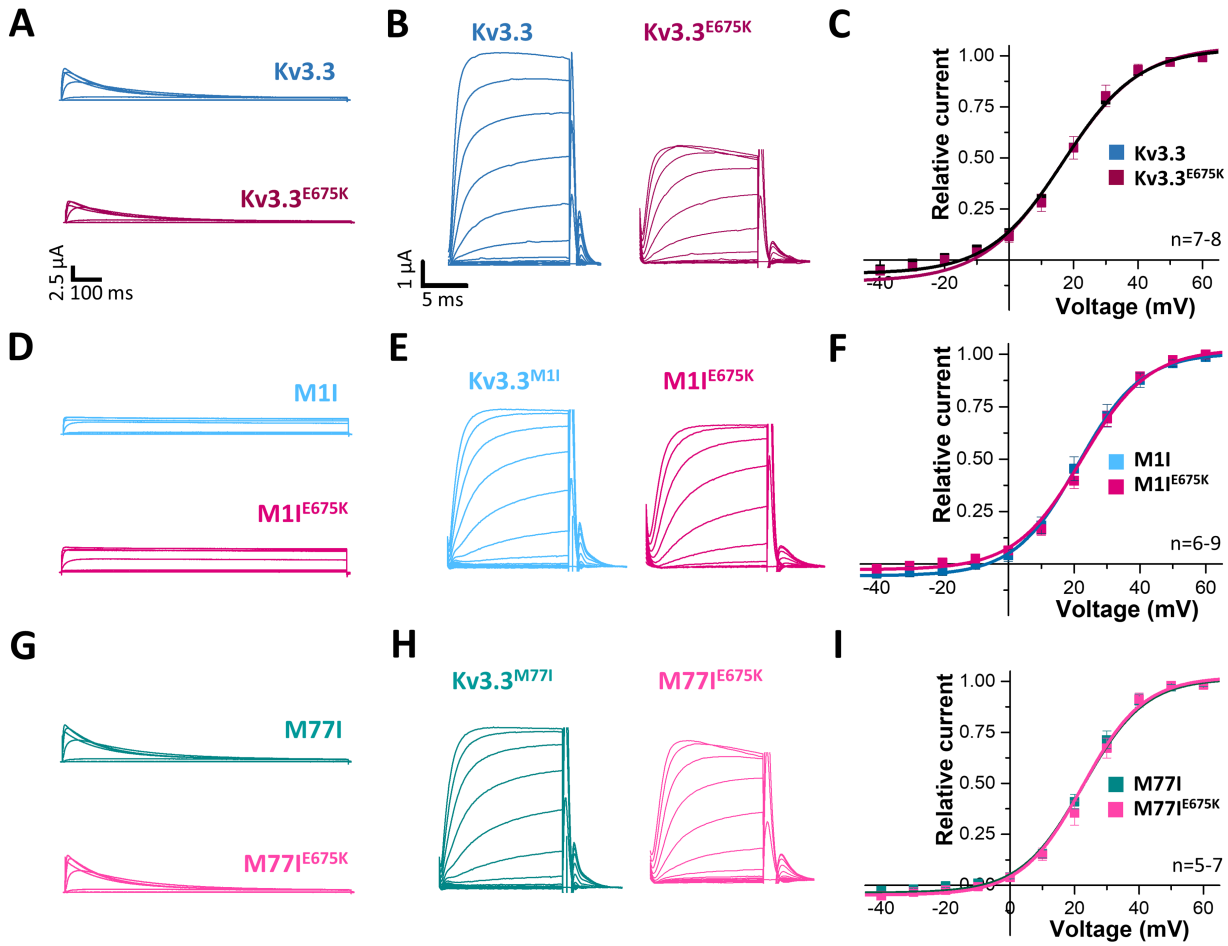
Voltage-dependence of activation of Kv3.3 and the Kv3.3^E675K^ variant in different channel backgrounds. **(A)** Representative current traces of wild-type Kv3.3 (top) and Kv3.3^E675K^ (bottom). The voltage protocol is illustrated in Supplementary Figure 5B. **(B)** Representative current traces using a short GV protocol (see Methods section and Supplementary Figure 5A) and **(C)** the conductance-voltage relationships derived from panel **(B)**. **(D–F)** The same as in panel **(A–C)**, but the E675K variant was studied in a forced short Kv3.3 channel (M1I mutant). **(G–I)** The same as in panel **(A–C)**, but the E675K variant was studied in the in a forced long Kv3.3 channel (M77I mutant). Data are presented as mean ± s.e.m. The number of replicates is indicated within the graphs.

To index analyze whether the variant might be disease-relevant, we compared the gating properties of the mutant voltage-gated channel with those of wild-type Kv3.3. Here, activation curves were recorded (Figure 2A) and the conductance-voltage relationships (GVs) of both channels were determined (Figures 2B,C). However, no differences between wild-type Kv3.3 and Kv3.3^E675K^ were observed, also reflected by nearly identical voltages of half-maximal activation (V_1/2_) and k-values. Interestingly, ATI can result in a Kv3.3 channel lacking the first 76 N-terminal amino acids and thus the so-called inactivation ball. Therefore, we also analyzed the voltage-dependence of the Kv3.3^E675K^ variant in both types of Kv3.3 channels. To this end, the short Kv3.3 channel background was generated by mutagenesis by removing the first start codon (M1I mutation) (Figures 1A, 2D–F) and the long channel variant by removing the second start codon (M77I) (Figures 1A, 2G–I). While both ATI variants showed a slightly shifted GV curve of about +5 mV toward more positive potentials (Supplementary Figure 2), the E675K variant did not lead to any further shift in the voltage-dependence in either the short (Figure 2F; Supplementary Figure 2) or the long Kv3.3 background (Figure 2I; Supplementary Figure 2). Moreover, activation and deactivation kinetics did not differ between the Kv3.3 wild-type channel and the Kv3.3^E675K^ variant (Supplementary Tables 3, 4).

**Figure 3:**
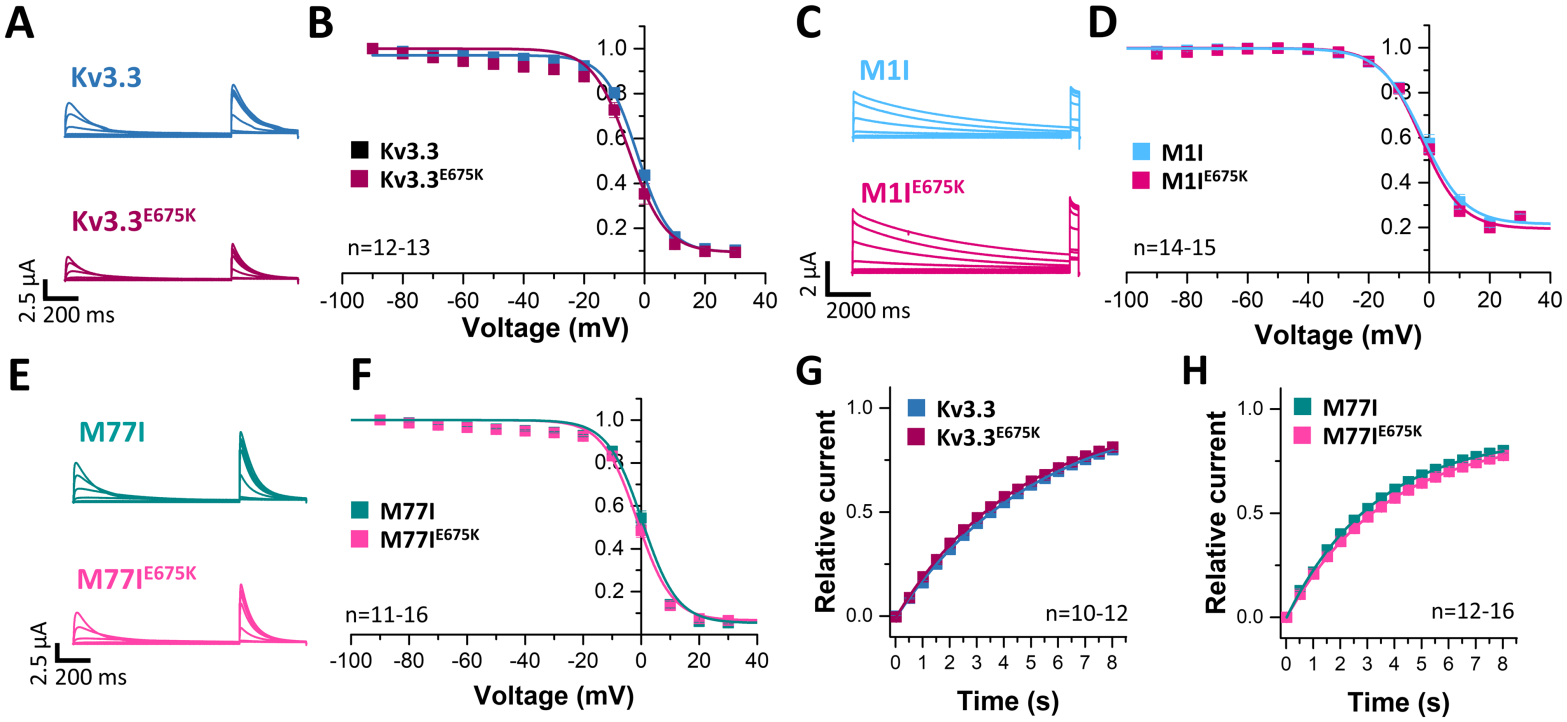
Voltage-dependence of inactivation and recovery from inactivation kinetics of Kv3.3 and the Kv3.3^E675K^ variant in different channel backgrounds. **(A)** Representative current traces of wild-type Kv3.3 (top) and Kv3.3^E675K^ (bottom) (The voltage protocol is illustrated in Supplementary Figure 5C) and **(B)** the derived inactivation curves. **(C,D)** The same as in panel **(A,B)**, but the E675K variant was studied in a forced short Kv3.3 channel (M1I mutant) (The voltage protocol is illustrated in Supplementary Figure 5D). **(E,F)** The same as in panel **(A,B)**, but the E675K variant was studied in the in a forced long Kv3.3 channel (M77I mutant). **(G)** Recovery from inactivation of wild-type Kv3.3 and Kv3.3^E675K^, fitted to a mono-exponential equation. The voltage protocol is illustrated in Supplementary Figure 5F. **(H)** The same as in panel **(G)**, but the E675K variant was studied in the in a forced long Kv3.3 channel (M77I mutant). Data are presented as mean ± s.e.m. The number of replicates is indicated within the graphs.

Next, we analyzed the voltage-dependent channel inactivation (Figures 3A–F) of the three different Kv3.3 channel constructs (wild-type, M1I, M77I) with and without the E675K variant. In the wild-type background (Figures 3A,B), the E675K variant did not alter the half-maximal voltage of inactivation (V_1/2_) or the k-values. As expected for a channel construct lacking the inactivation ball, the M1I Kv3.3 channel exhibits a reduced steady-state inactivation (Supplementary Figures 3A,C), and an increased k-value (Supplementary Figure 3C). However, in the long M77I Kv3.3 channel the inactivation parameters were not altered compared to wild-type Kv3.3 (Supplementary Figure 3), indicating that the long channel is the predominantly translated channel variant in our expression system. Similar as in the wild-type background (Figures 3A,B), the E675K variant did not lead to any changes in the inactivation properties compared to the channels in the M1I (Figures 3C,D) or M77I background (Figures 3E,F).

Also the recovery from inactivation was comparable for wild-type Kv3.3 and the Kv3.3^E675K^ variant, as well as for the M77I variant with and without the E675K exchange, respectively (Figures 3G,H). Note, that this parameter was not analyzed in the Kv3.3^M1I^ background, as this variant does not show a fast inactivation *per se*.

### 3.6 The Kv3.3^E675K^ variant does not interfere with the TBK1 modulation

As mentioned above, a mutation in the Kv3.3 channel (Kv3.3^G592R^) was reported to cause SCA13 by enhancing the activation of TBK1. TBK1 activity is required for the binding of Kv3.3 to its auxiliary subunit Hax-1, which prevents channel inactivation. Consequently, the application of the TBK1 inhibitor MRT67307 increases Kv3.3 channel inactivation by preventing Hax-1 binding and channel inactivation (Zhang et al., 2021b). Thus, we investigated, whether the Kv3.3^E675K^ variant has an impact in this mechanism.

**Figure 4:**
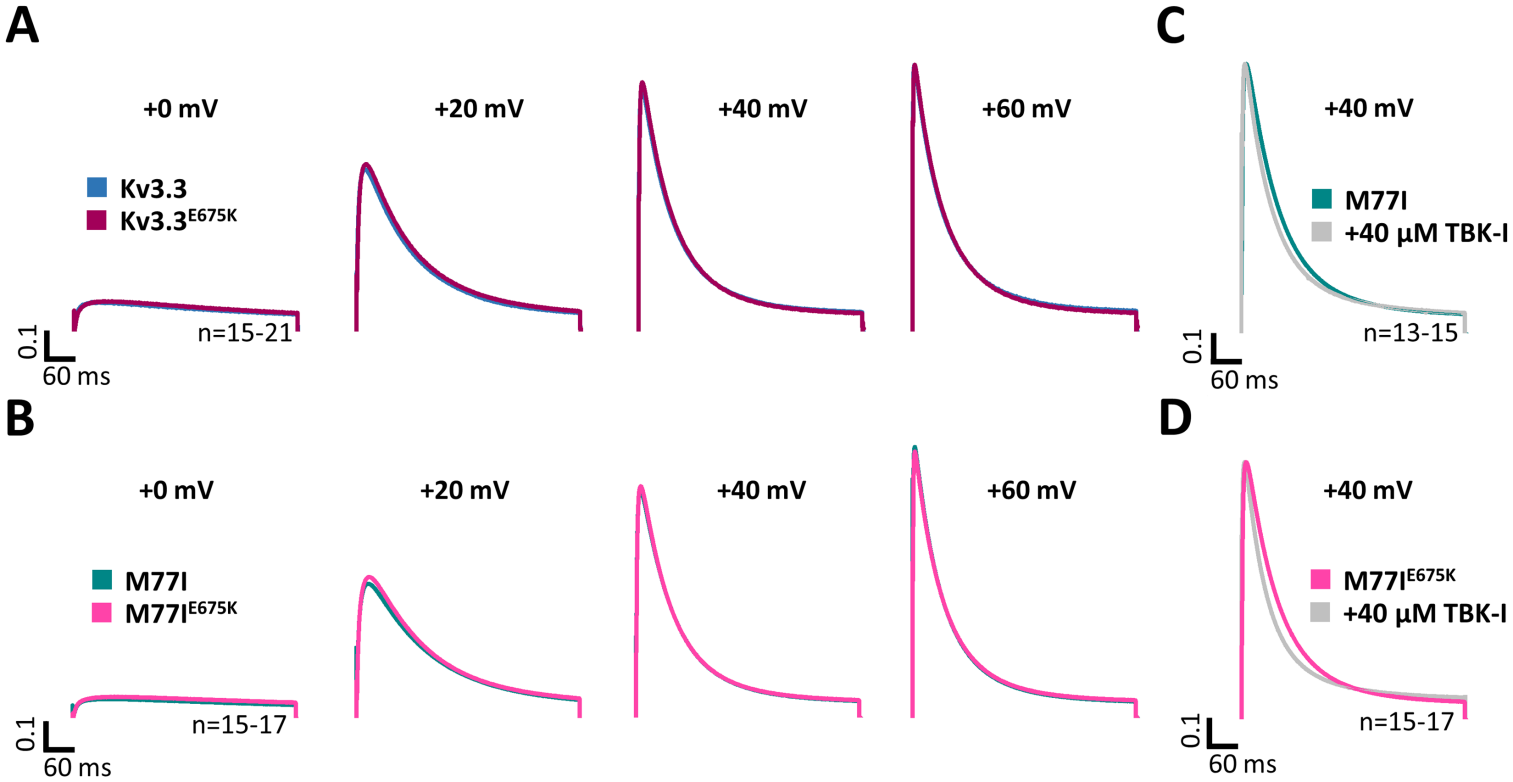
Inactivation kinetics and TBK1 modulation of Kv3.3 and the Kv3.3^E675K^ variant. **(A)** Averaged current traces to illustrate the voltage-dependence of activation and the inactivation kinetics at different potentials for wild-type Kv3.3 and Kv3.3^E675K^ (The voltage protocol is illustrated in Supplementary Figure 5B). **(B)** Similar as in panel **(A)** but the E675K variant was studied in the in a forced long Kv3.3 channel (M77I mutant). **(C)** Averaged current traces to illustrate the inactivation kinetics of the forced long Kv3.3 channel (M77I mutant) without or after pre-treatment with a TBK1 inhibitor. The time constants of inactivation of the M77I Kv3.3 channel after TBK1 inhibitor treatment were: *τ*_1_ = 144 ms, τ_2_ = 50 ms, A1/(A1 + A2) = 0.3. **(D)** The same as in panel **(C)**, but for the forced long variant carrying the E675K variant. The time constants of inactivation of the M77I Kv3.3^E675K^ variant after TBK1 inhibitor treatment were: τ_1_ = 136, τ_2_ = 43, A1/(A1 + A2) = 0.3. The number of replicates is indicated within the graphs.

Here, we first compared the inactivation kinetics of wild-type Kv3.3 and Kv3.3^E675K^ at different potentials in more detail (Figure 4A), to probe for a loss-of-function by a change in the basal inactivation kinetics, however, no differences were observed. Also in the long M77I channel background, the E675K variant has no impact on the inactivation kinetics (Figure 4B). These data suggest that Hax-1 binding is not altered in the Kv3.3^E675K^ variant. Next, we incubated the Kv3.3 cRNA-injected oocytes before the electrophysiological measurements in recording solution containing the TBK1 inhibitor MRT67307. For these experiments, we studied the forced long M77I construct to ensure that all channels at the plasma membrane harbor an inactivation ball. As expected, TBK1 inhibition accelerated the rate of inactivation of the long M77I channel construct (Figure 4C). However, also for the E675K variant a highly similar effect, with a small acceleration of the rate of inactivation, was observed after the pre-treatment with the TBK1 inhibitor (Figure 4D).

### 3.7 Kv3.3^E675K^ reduces the current amplitude and has no dominant-negative effect on wild-type Kv3.3 channels

**Figure 5:**
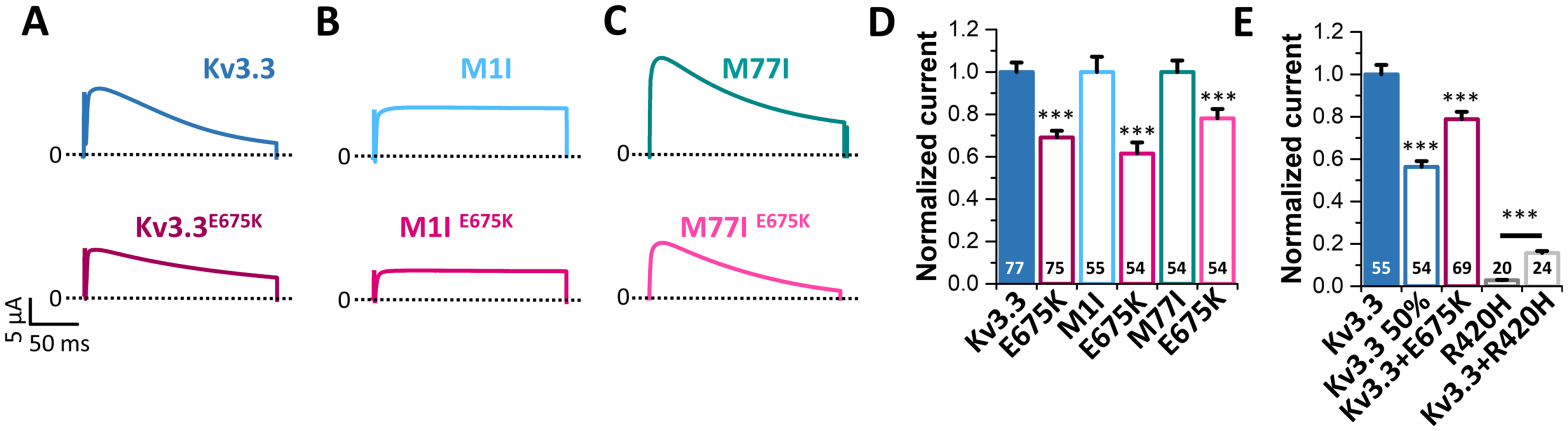
The Kv3.3^E675K^ variant reduces the current amplitudes of Kv3.3 channels. **(A)** Representative current traces of Kv3.3 and the E675K variant introduced in wild-type Kv3.3. The voltage protocol is illustrated in Supplementary Figure 5E. **(B)** The same as in panel **(A)** but the E675K variant was introduced in the forced short M1I Kv3.3 channel or **(C)** the forced long M77I Kv3.3 channel background. **(D)** Peak current amplitude analyses derived from panel **(A–C)**. Plotted are the current amplitudes of the respective Kv3.3 channel constructs, in the absence or presence of the E675K variant. Currents were analyzed after a voltage step to +50 mV, 2 days after injection of 0.5 ng cRNA of the respective channel construct into each oocyte. Currents were normalized to the respective Kv3.3 channel background. **(E)** Oocytes were injected with 0.5 ng (Kv3.3) or to mimic a haploinsufficiency with 0.25 ng (Kv3.3 50%) wild-type Kv3.3 cRNA. To mimic the heterozygous state of the patient, 0.25 ng wild-type Kv3.3 was co-injected with 0.25 ng Kv3.3^E675K^ cRNA (Kv3.3 + E675K). As a comparison to a SCA13 mutant, we injected 0.5 ng cRNA of the dominant-negative Kv3.3^R420H^ mutant (Kv3.3^R420H^) alone or 0.25 ng wild-type Kv3.3 together with 0.25 ng Kv3.3^R420H^. Currents were analyzed as in panel **(D)** and normalized to wild-type Kv3.3. Data are presented as mean ± s.e.m. The number of replicates is indicated within the graphs.

Since voltage-dependence and current kinetics were not altered by the E675K variant, we probed for changes in the macroscopic current amplitude as a possible disease-causing mechanism. To this end, similar amounts of cRNA were injected for the Kv3.3, M1I, M77I channel constructs with or without the E675K exchange and peak current amplitudes were analyzed (Figure 5). Strikingly, the E675K variant reduced the current amplitudes by approximately 30% in all Kv3.3 channel backgrounds (Figures 5A–D). As the patient is a heterozygous mutation carrier, we also analyzed the current amplitudes in the heterozygous state by co-injecting half the amount of wild-type Kv3.3 with the Kv3.3^E675K^ (Figure 5E). Similarly to the condition reflecting the homozygous state (Figures 5A–D), Kv3.3^E675K^ reduced the current amplitudes compared to wild-type Kv3.3 (Figure 5E). Strikingly, this current reduction was not dominant-negative, as observed for the SCA13 causing Kv3.3^R420H^ mutation (Waters et al., 2006) (Figure 5E). Note, that the patient described in the current study has a mild cerebellar ataxia with a dominant central vertigo and no classical SCA13, which may be reflected by this mechanistic difference between the two mutants.

### 3.8 Pharmacological approaches to rescue Kv3.3^E675K^ channel loss-of-function

**Figure 6:**
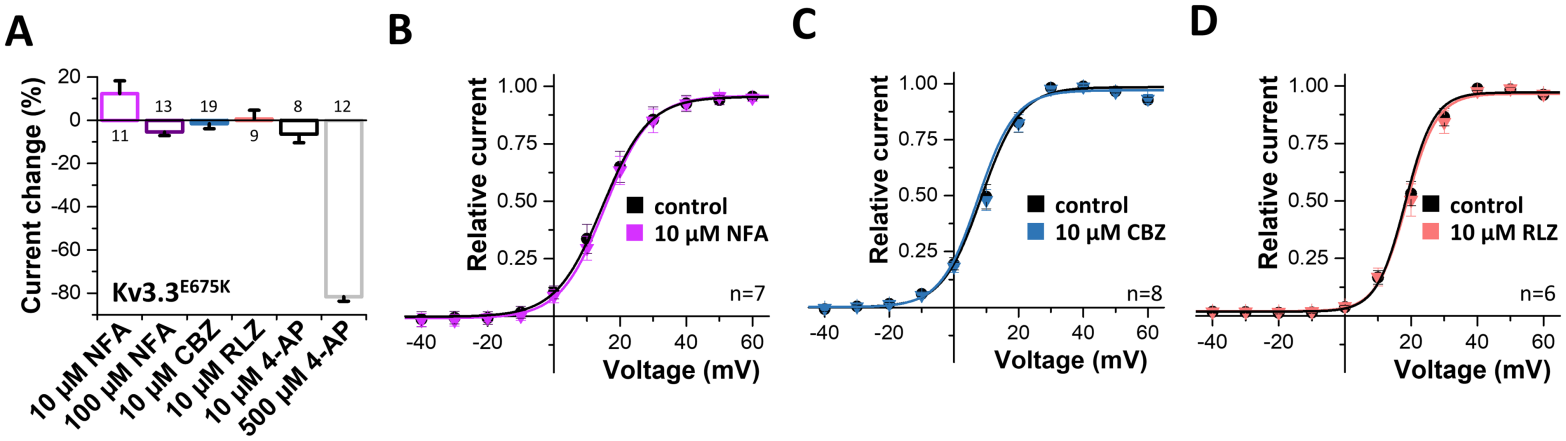
Search for drugs to rescue the Kv3.3^E675K^-mediated loss-of-function. **(A)** Analysis of the current changes of the Kv3.3^E675K^ variant by the application of niflumic acid (NFA), carbamazepine (CBZ), riluzole (RLZ) or 4-AP, analyzed at +40 mV. **(B)** Conductance-voltage relationships before and after perfusion with 10 μM niflumic acid, **(C)** 10 μM carbamazepine or **(D)** 10 μM riluzole. The voltage protocol is illustrated in Supplementary Figure 5A. Data are presented as mean ± s.e.m. The number of replicates is indicated within the graphs.

Next, we probed for drugs to rescue Kv3.3 channel function. As Kv3.3^E675K^ leads to reduced current amplitudes in the homozygous as well as in the heterozygous state, we applied different compounds in order to activate Kv3.3 (Supplementary Figure 4) and Kv3.3^E675K^ (Figure 6). First, we tested the Kv1.1 activator niflumic acid (Servettini et al., 2023), however, only a slight, non-significant increase in Kv3.3^E675K^ channel amplitude was observed at a concentration of 10 μM (Figures 6A,B). Unfortunately, even at 100 μM, a concentration that is most likely too high to be achieved in the plasma of patients, application of niflumic acid resulted only in a minimal non-significant decrease in currents of wild-type Kv3.3 (Supplementary Figure 4A) or the Kv3.3^E675K^ variant (Figure 6A). Carbamazepine that is used in the treatment of epilepsy did not activate Kv3.3 (Figures 6A,C). On the other hand, 4-AP, as expected for an unspecific Kv channel blocker, caused an inhibition of Kv3.3^E675K^, albeit only in high concentrations (Figure 6A). Noteworthy, no improvement was noted by 4-AP treatment in the patient. In contrast, the symptoms increased dramatically under 4-AP treatment which is consistent with the hypotheses that reduced Kv channel amplitudes represent the disease-causing mechanism in our patient. Therefore, we next tested riluzole a drug that is used to treat amyotrophic lateral sclerosis (ALS), since it is known to activate potassium channels, e.g., the K_2P_ channels TREK-1 and TRAAK (Duprat et al., 2000) or small-conductance Ca^2+^ activated potassium channels (Cao et al., 2002). However, no activation of Kv3.3^E675K^ was observed (Figures 6A,D). Note, that riluzole, known to activate other potassium channels, showed a positive effect on symptom severity and frequency of manifestation in our patient, which might be caused by a rescue of collective potassium conductance. All tested drugs showed similar effects on wild-type Kv3.3 channels (Supplementary Figure 4).

### 3.9 Extracellular potassium ions increase the current density of wild-type and mutant Kv3.3 channels

**Figure 7:**
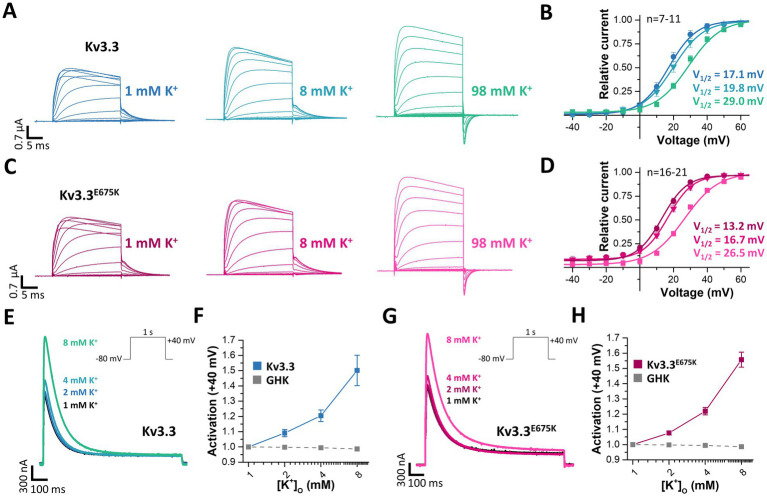
Rescue of Kv3.3 and Kv3.3^E675K^, by increased peak current amplitudes and reduced channel inactivation, mediated by [K^+^]_ex_. **(A)** Representative conductance-voltage (GV) recordings of Kv3.3 at different [K^+^]_ex_ and **(B)** the derived GV relationships, analyzed from the tail currents at different [K^+^]_ex_. **(C,D)** Similar as in panel **(A,B)** but for the Kv3.3^E675K^ variant. The voltage protocol is illustrated in Supplementary Figure 5A with a step to −15mV, −25mV and −40 mV, respectively. **(E)** A voltage step from −80 to +40 mV (see inset) was applied to record channel activation and peak currents of wild-type Kv3.3 at different [K^+^]_ex_. **(F)** Analysis of wild-type Kv3.3 channel activation at +40 mV by different [K^+^]_ex_. As a comparison to illustrate the paradoxical Kv3.3 activation by [K^+^]_ex_, the expected current changes, according to the Goldmann-Hodgkin-Katz equation, are illustrated as dotted line. **(G,H)** Similar as panel **(E,F)** but for the Kv3.3^E675K^ variant. Data are presented as mean ± s.e.m. The number of replicates is indicated within the graphs.

Voltage-gated potassium channels, like hERG (Kv11.1) (Guo et al., 2009), or Kv7.1 (Abrahamyan et al., 2023) or inward rectifier potassium channels (Leech and Stanfield, 1981) are modulated by extracellular potassium ions ([K^+^]_ex_). Thus, we aimed to analyze, whether Kv3.3 is also dependent on [K^+^]_ex_, as channel activation by extracellular potassium might rescue the channel loss-of-function induced by the E675K variant. Indeed, a current increase by extracellular potassium ions was observed for wild-type Kv3.3 channels and Kv3.3^E675K^ accompanied by a loss of channel inactivation (rectification) at depolarized potentials (Figure 7A). We also found that the GV curve was shifted to more depolarized potentials by increasing [K^+^]_ex_. However, a putative current reduction at potentials between +10 to +40 mV, caused by this rightward shift in the voltage-dependence by increased [K^+^]_ex_, was only observed at extremely high [K^+^]_ex_ (Figure 7B). As the Kv3.3^E675K^ variant showed a similar [K^+^]_ex_-dependence (Figures 7C,D), increasing extracellular potassium should result in a current increase for both, wild-type Kv3.3 and the Kv3.3^E675K^ variant if [K^+^]_ex_ concentrations are not far too supra-physiological (e.g., 8 mM).

Therefore, we next analyzed the peak current amplitudes of both, Kv3.3 and Kv3.3^E675K^, by increasing [K^+^]_ex_ in the range of 1–8 mM (Figures 7E,G). Unlike expected from the Goldman-Hodgkin-Katz equation, an apparently paradoxical increase in outward currents was observed for both, wild-type Kv3.3 (Figures 7E,F) and the Kv3.3^E675K^ variant (Figures 7G,H). Thus, Kv3.3 and Kv3.3^E675K^ channels are indeed activated by increasing [K^+^]_ex_ and consequently increasing [K^+^]_ex_ might be in theory an option to rescue channel function if sufficient effects also occur in the physiological range.

### 3.10 Extracellular potassium ions destabilize inactivation of both, wild-type and Kv3.3^E675K^ channels

**Figure 8:**
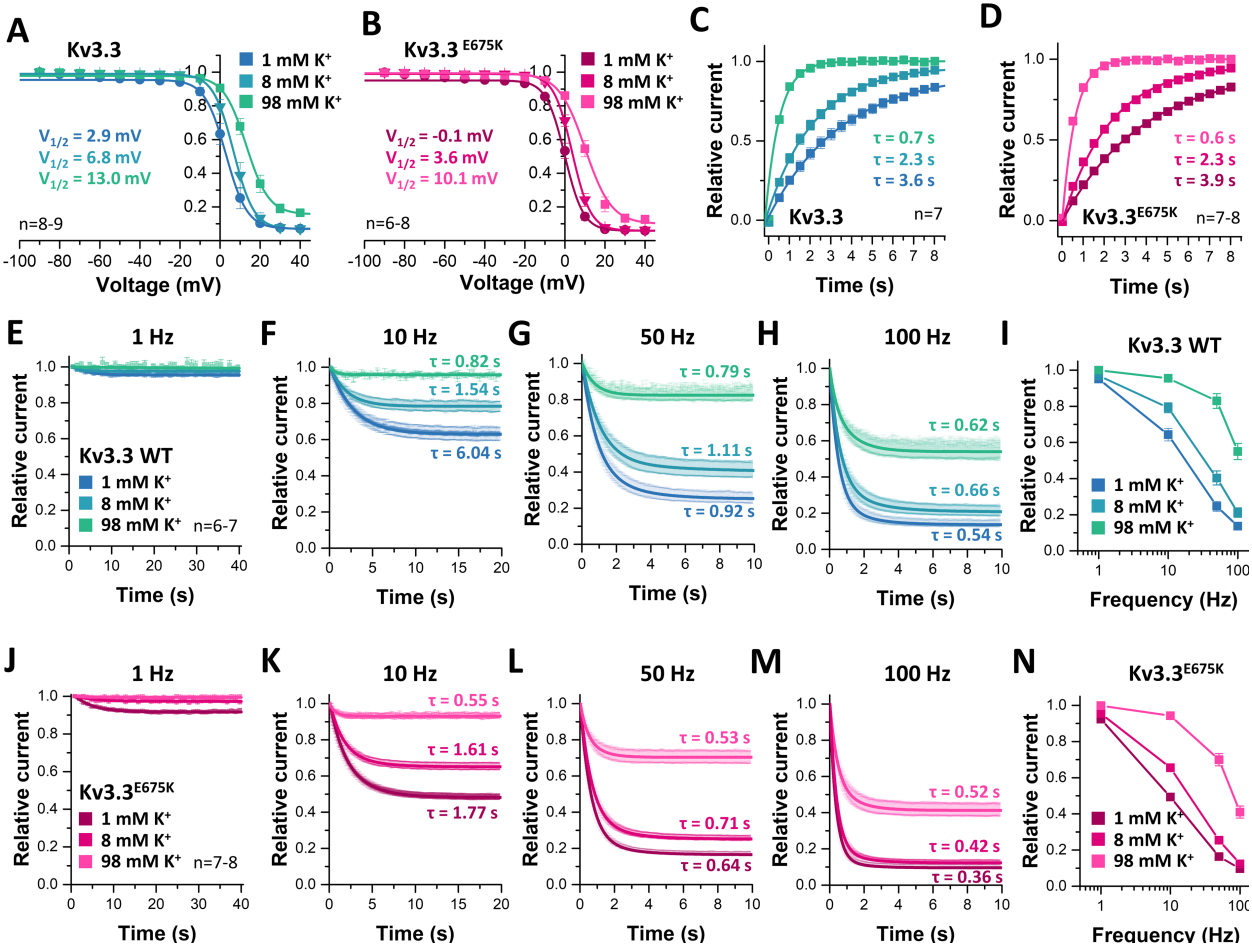
Rescue of Kv3.3 and Kv3.3^E675K^, by a shift in the voltage-dependence of inactivation, speeding of the recovery from inactivation and a reduced cumulative inactivation, mediated by [K^+^]_ex_. Inactivation curves of **(A)** Kv3.3 or **(B)** Kv3.3^E675K^ at different [K^+^]_ex_. The V_1/2_ of inactivation for the different [K^+^]_ex_ are provided within in the graphs. The voltage protocol is illustrated in Supplementary Figure 5C. **(C)** Recovery from inactivation of Kv3.3 or **(D)** Kv3.3^E675K^ at different [K^+^]_ex_. The time constants τ for the recovery from inactivation are calculated from the mono-exponential fits illustrated within the graphs. The voltage protocol is illustrated in Supplementary Figure 5F. Cumulative inactivation of Kv3.3 **(E–I)** and Kv3.3^E675K^ **(J–N)** at **(E,J)** 1 Hz, **(F,K)** 10 Hz, **(G,L)** 50 Hz, and **(H,M)** 100 Hz recorded at different [K^+^]_ex_. The indicated time constants τ for the first 2 s of cumulative inactivation are calculated from mono-exponential fits. The voltage protocol is illustrated at Supplementary Figure 5G. **(I,N)** Relative currents of Kv3.3 **(I)** and Kv3.3^E675^K **(N)** after 10 s of pulsing with different frequencies at different [K^+^]_ex_, normalized to the current amplitude of the first pulse. Data are presented as mean ± s.e.m. The number of replicates is indicated within the graphs.

As we observed a loss of channel inactivation at depolarized potentials at elevated [K^+^]_ex_ (Figure 7A), we studied the inactivation properties in relation to [K^+^]_ex_ (Figure 8). The voltage of half-maximal inactivation was shifted rightwards by increasing [K^+^]_ex_ for both, wild-type (Figure 8A) and mutant Kv3.3^E675K^ channels (Figure 8B). The pronounced potassium-dependent increase in half-maximal voltage of inactivation confirms a [K^+^]_ex_-induced destabilization of channel inactivation and might rescue channel function *in vivo* via this mechanism.

Next, we analyzed the recovery from inactivation in relation to [K^+^]_ex_. Both, wild-type (Figure 8C) and mutant Kv3.3^E675K^ (Figure 8D) channels showed a faster recovery from inactivation by increasing [K^+^]_ex_, again demonstrating a destabilization of the inactivation by [K^+^]_ex_. Also this effect might mechanistically contribute to rescue Kv3.3^E675K^ channel function. Note that the calculated time constants of the recovery from inactivation for wild-type Kv3.3 and the Kv3.3^E675K^ variant at different [K^+^]_ex_ were not significantly altered.

**Figure 9:**
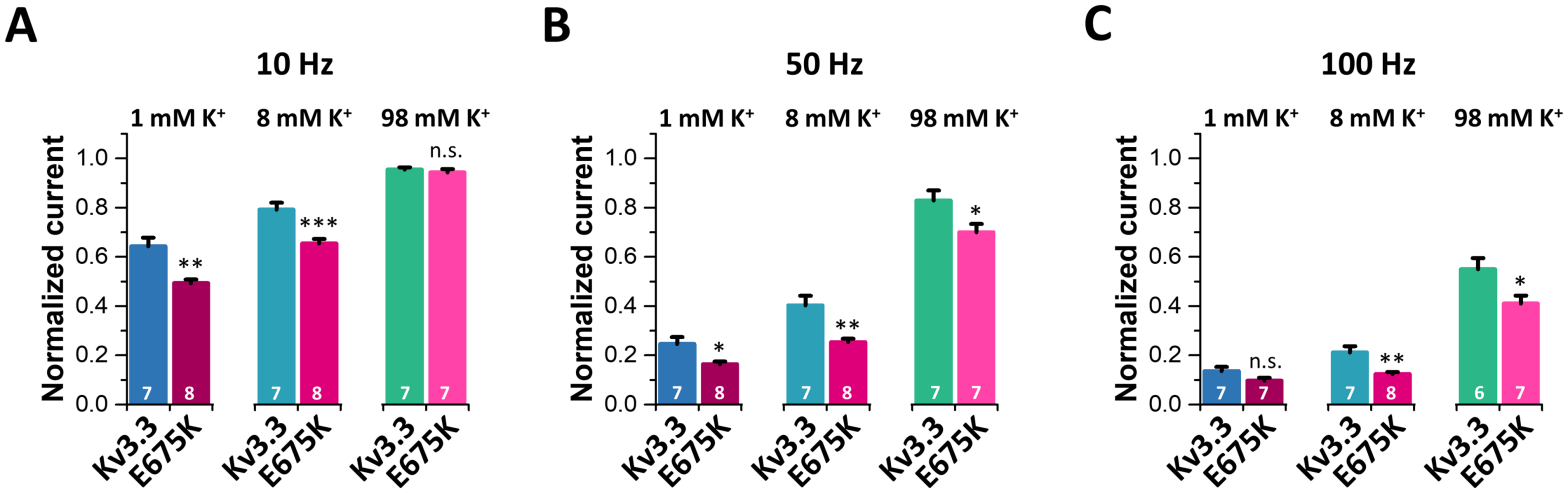
Analyses of the frequency-dependent accumulation of inactivation for Kv3.3 and Kv3.3^E675K^ at different frequencies and [K^+^]_ex_. The data were analyzed from recordings illustrated in Figure 8. Relative current amplitudes after 10 s of pulsing at different [K^+^]_ex_ (1 mM, 8 mM, 98 mM) at 10 Hz **(A)**, 50 Hz **(B)** and 100 Hz **(C)**. The current was normalized to the amplitude of the first pulse. Data are presented as mean ± s.e.m. The number of replicates is indicated within the graphs.

As cerebellar neurons have a high spiking frequency and [K^+^]_ex_ reduces inactivation, we next probed the cumulative inactivation of Kv3.3 (Figures 8E–I) and the Kv3.3^E675K^ variant (Figures 8J–N) at different frequencies and [K^+^]_ex_. As expected, the cumulative inactivation of wild-type Kv3.3 (Figures 8E–I) and of Kv3.3^E675K^ (Figures 8J–N) increases with elevated frequencies between 1 and 100 Hz. Strikingly, increasing [K^+^]_ex_ leads to a reduced cumulative inactivation of Kv3.3^E675K^ which was most pronounced at frequencies between 10 and 50 Hz (Figures 8J–N), an effect that might again rescue Kv3.3^E675K^ channel loss-of-function in the patient. Noteworthy, the accumulation of inactivation was across all tested frequencies more pronounced in the Kv3.3^E675K^ variant (Figures 8J–N) compared to wild-type Kv3.3 (Figures 8E–I). These effects are quantified and summarized in Figure 9 and the Supplementary Table 5. In addition, the speed of cumulative inactivation was also enhanced in the Kv3.3^E675K^ variant (Figures 8J–N) compared to wild-type Kv3.3 (Figures 8E–I), which is reflected by smaller time constants *τ* of the developing cumulative inactivation indicted within the graphs. The enhanced and more pronounced cumulative inactivation described here is likely to contribute to the pathophysiological loss-of-function of the Kv3.3^E675K^ variant.

As similar effects of extracellular potassium on cumulative inactivation were observed for wild-type Kv3.3 channels (Figures 8E–I, 9), increasing extracellular potassium might rescue overall Kv3.3 channel function in the patient, independent of whether [K^+^]_ex_ targets the Kv3.3^E675K^ mutant or wild-type channels. However, it is indispensable to determine the precise benefit of subtle alterations of [K^+^]_ex_ within the physiological range *in vivo*, to define a biological effectiveness for the patients.

## 4 Discussion

In the present study, we describe an atypical case of SCA13 with a heterozygous non-dominant negative loss-of-function variant in Kv3.3. In this atypical case, the symptoms of ataxia were mild, with a lack of cerebellar atrophy, while the rotational vertigo was the predominant phenotype and a major source of distress. The central origin of rotational vertigo, in particular through Kv3.3 channels, can be elucidated by considering the role of these potassium channels in the regulation of neuronal excitability and synaptic transmission within the central nervous system. Dysregulation of Kv3.3 channels, which are known for their fast activation and inactivation (Rudy and McBain, 2001; Kaczmarek and Zhang, 2017; Richardson et al., 2022), as well as their high expression in neurons involved in auditory and vestibular (Brooke et al., 2010) processing, may affect the firing patterns of neurons responsible for processing vestibular signals. This perturbation may lead to changes in the perception of head movements and spatial orientation, ultimately contributing to the manifestation of rotational vertigo of central origin (Hurlock et al., 2009; Kolkman et al., 2011; Middlebrooks et al., 2013).

The differences in phenotypes observed in the patient compared to those with classical SCA13 mutations (Waters, 1993) may be related to the mechanism of the observed loss-of-function in Kv3.3. Classical SCA13 mutations in the Kv3.3 channel act in a dominant-negative manner and/or show shifted activation voltages and/or result in increased activation of TBK1 (Zhang et al., 2021a). For example, overactivation of TBK1 by Kv3.3^G592R^ results in a loss of Hax-1 binding to the channel (Zhang et al., 2021b), ultimately leading to increased rates of cell death, which may acting the cerebellar atrophy (Waters, 1993). In contrast, the Kv3.3^E675K^ variant described here does not interfere with the TBK1 regulation and the patient does not show a cerebellar atrophy. The differences in the phenotypic spectrum may be due to the fact that the novel variant described here induces a loss-of-function which is not acting in a dominant-negative manner or by a shift in the voltage-dependence, as in the previously described Kv3.3 variants (Supplementary Table 1). Strikingly, although we did not observe a cerebellar atrophy, morphological changes due to global brain volume loss were observed by MRI. We did not observe any ß-amyloid-or tau pathology in the CSF. However, the MRI data together with the neuropsychological observations are consistent with the development of an early stage of Alzheimer’s disease or another yet to be described form of neurodegenerative mental decline. Strikingly, a decrease in Kv3 currents was previously proposed to play a role in Alzheimer’s disease (Franciosi et al., 2006; Pannaccione et al., 2007; Boda et al., 2012).

In our experiments, we observed a relatively minor effect of TBK1 inhibition on Kv3.3 channel inactivation kinetics compared to what has been described by Zhang et al. (2021b) which indicates that the effects depend on the heterologous expression system and maybe interactions with the respective actin cytoskeleton. Since the inactivation particle of the Kv3.3 channel has been shown to interact with the actin-Arp2/3-Hax-1 complex and TBK1 has been proposed to interact with the N-terminus (Zhang et al., 2016; Zhang et al., 2021b), we studied the effects of the E675K variant in the full length Kv3.3^M77I^ construct, as here the N-terminus cannot be removed by ATI. However, as it was already hypothesized that the TBK1/Hax-1 regulation is not only mediated by the N-terminal inactivation particle (Zhang et al., 2021b), we also tested the effect of the E675K variant on TBK1 modulation in the Kv3.3^M1I^ channel background, lacking the N-terminal inactivation ball (Supplementary Figure 6). However, the TBK1 inhibition resulted in an even more pronounced acceleration of the inactivation kinetics, which was similar for the “wild-type” Kv3.3^M1I^ and the Kv3.3^M1I/E675K^ variant, supporting that the TBK1 modulation is not exclusively mediated by the N-terminus (Zhang et al., 2021b) and not influenced by the variant. The lack of effects of the E675K variant on TBK1 modulation is in agreement with previous co-immunoprecipitation experiments, where the entire C-terminus of the Kv3.3 channel could be deleted following the residue 654 without interfering with a TBK1 co-immunoprecipitation (Zhang et al., 2021b).

Since Kv3.3^E675K^ results in reduced current amplitudes in both the homozygous and the heterozygous state, a personalized drug approach should aim to either rescue the reduced Kv3.3 current amplitudes or increase the potassium conductance of another potassium channel to replace the Kv3.3^E675K^ loss-of-function. Consistent with this theory and that a Kv channel loss-of-function underlies the phenotypic spectrum in our patient, an additional reduction of Kv currents, as attempted in the early stages of the clinical evaluation and treatment of the patient, increased the severity of the symptoms. Current therapy focuses on antiepileptic drugs such as carbamazepine and riluzole, which may antagonize the increase in excitability caused by reduced potassium currents via an inhibition of sodium channels. Strikingly, riluzole, which is used to treat ALS, was the most effective drug for the attenuation of symptoms. Although riluzole does not act directly on Kv3.3 channels, it is a “notorious” pan-potassium channel activator (Lauxmann et al., 2021), activating for example the K_2P_ channels TREK-1 and TRAAK (Duprat et al., 2000) or small-conductance Ca^2+^-activated potassium channels (Cao et al., 2002). This potassium channel activation could replace the Kv3.3^E675K^ loss-of-function, while any remaining hyperexcitability is dampened by its ability to block calcium-and sodium channels (Stefani et al., 1997) and/or glutamate signaling (Couratier et al., 1994; Doble, 1996; Kretschmer et al., 1998). Due to the pleiotropic pharmacological characteristics of riluzole, it is obviously difficult to identify the exact pharmacological mode of action using a “simplified” heterologous expression system to explain the *in vivo* effectiveness of the drug in our patient.

Although we provide compelling evidence that non-dominant negative loss-of-function variants in *KCNC3* lead to an atypical form of SCA13, it is currently not possible to definitively prove the causality of this variant. Certainly, we cannot exclude that other variants in the genome of the patient contribute to the phenotype in our case, or that additional epigenetic factors are involved to ultimately trigger the severe vertigo phenotype. A family with more carriers of the Kv3.3^E675K^ variant, which would allow testing for a good genotype–phenotype correlation, would shed light on whether this exchange causes atypical SCA13 in a monogenetic trait and on the penetrance of vertigo in patients with this variant. Nevertheless, our study clearly associated non-dominant negative loss-of-function variants in *KCNC3* with atypical SCA13, in which other phenotypes such as vertigo may dominate the clinical features.

Our observation that extracellular potassium increases the Kv3.3 outward currents and that it reduces cumulative Kv3.3 inactivation by destabilizing the inactivated state of the channel provides a promising therapeutic approach to treat the patient, but also other SCA13 cases with Kv3.3 loss-of-function mutations. However, further research is needed to investigate the mechanisms behind this [K^+^]_ex_-dependent Kv3.3 modulation. In our experimental setting, elevated potassium concentrations were accompanied by reduced sodium concentrations (Na^+^ replaced by K^+^), the observed [K^+^]_ex_-dependent gain-of-function could mechanistically result from attenuation of an extracellular sodium block, similar to what has been described for the hERG (Kv11.1) channel (Numaguchi et al., 2000). This putative competition between extracellular sodium and potassium may be of interest for future experiments on the Kv3.3 channel modulation by extracellular cations. Following the experimental part of the study, we proposed that the patient be given potassium supplementation in a carefully controlled manner to see if this would lead to an improvement in symptoms. Alternatively, since it is not reasonable to increase extracellular potassium to supraphysiological concentrations, it is promising to develop a new class of drugs that destabilize Kv3.3 inactivation, thus acting as extracellular potassium mimetics.

In conclusion, our study increases the diversity of pathogenic potassium channel variants in neurological disorders. The functional analysis performed together with the experiments exploring a personalized pharmacological approach to rescue potassium and/or Kv3.3 conductance provide an important first step toward a personalized treatment of patients with atypical, but maybe also with common, SCA13 variants.

## Data Availability

The original contributions presented in the study are included in the article/Supplementary material, further inquiries can be directed to the corresponding author.

## Footnotes

^1^ https://www.washington.edu/

^2^ http://gnomad-sg.org/

^3^ https://www.ebi.ac.uk/jdispatcher/msa/clustalo
