# Supplementary material for: A novel KCNC3 gene variant in the voltage-dependent Kv3.3 channel in an atypical form of SCA13 with dominant central vertigo
Source: Front Cell Neurosci. 2024 Oct 2;18:1441257. doi: 10.3389/fncel.2024.1441257 (PMC11480015; doi:10.3389/fncel.2024.1441257)
Supplement: Supplementary file 1 [file Data_Sheet_1.PDF]

Supplementary Material

1 Supplementary Figures and Tables

1.1 Supplementary Figures

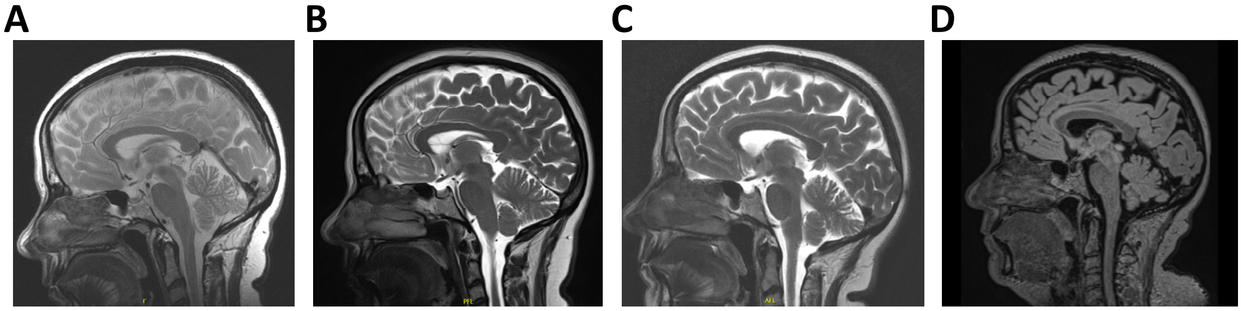

Supplementary Figure 1. Mid-sagittal T1/T2 and Flair-sequence MR images. Images were taken in **(A)** 2010, **(B)** 2014, **(C)** 2019, and **(D)** 2024, respectively.

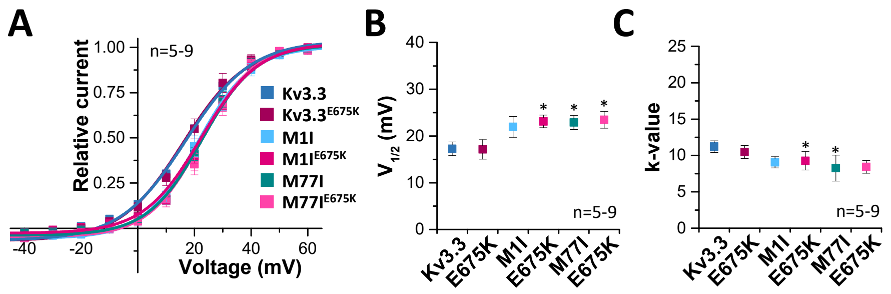

Supplementary Figure 2. Voltage-dependence of activation for the E675K variant studied in all three Kv3.3 channel backgrounds. **(A)** Conductance-voltage relationships, **(B)** values for the voltages of half-maximal activation ($V_{1/2}$) and **(C)** the k-values. Data are presented as mean ± s.e.m.. The number of replicates is indicated within the graphs. n.s., not significant. *, $p < 0.05$; **, $p < 0.01$; ***, $p < 0.001$.

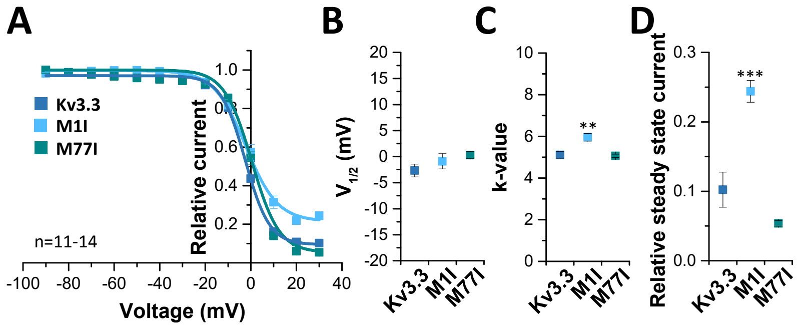

Supplementary Figure 3. Voltage-dependence of inactivation of all three Kv3.3 channel constructs. **(A)** Voltage-dependence of inactivation, **(B)** the voltages of half-maximal inactivation ($V_{1/2}$) and **(C)** the k-values. **(D)** Analysis of the steady-state inactivation, plotting the sustained currents. Data are presented as mean ± s.e.m.. The number of replicates is indicated within the graphs. n.s., not significant. *, $p < 0.05$; **, $p < 0.01$; ***, $p < 0.001$.

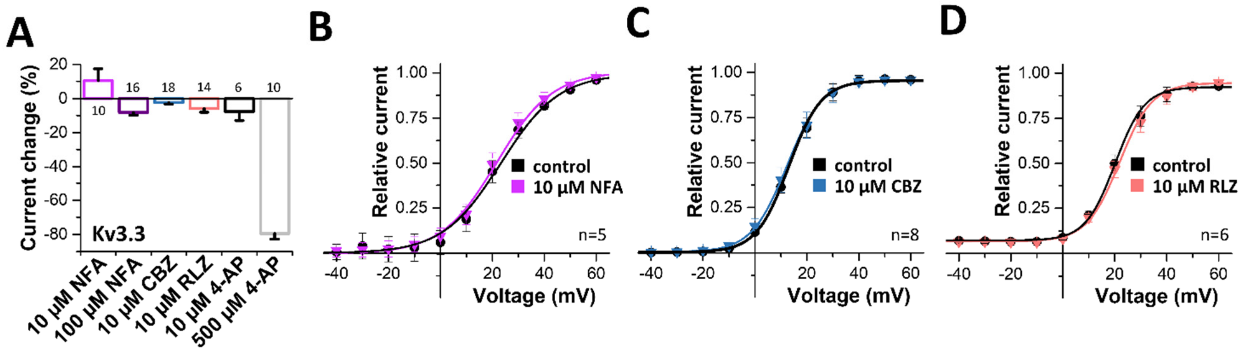

Supplementary Figure 4. Search for drugs to increase wild-type Kv3.3 currents. **(A)** Analysis of the peak current changes of wild-type Kv3.3 by the application of niflumic acid (NFA), carbamazepine (CBZ), riluzole (RLZ) or 4-AP, analyzed at +40 mV. **(B)** Conductance-voltage relationships before and after perfusion with 10 µM niflumic acid, **(C)** 10 µM carbamazepine or **(D)** 10 µM riluzole. Data are presented as mean ± s.e.m.. The number of replicates is indicated within the graphs.

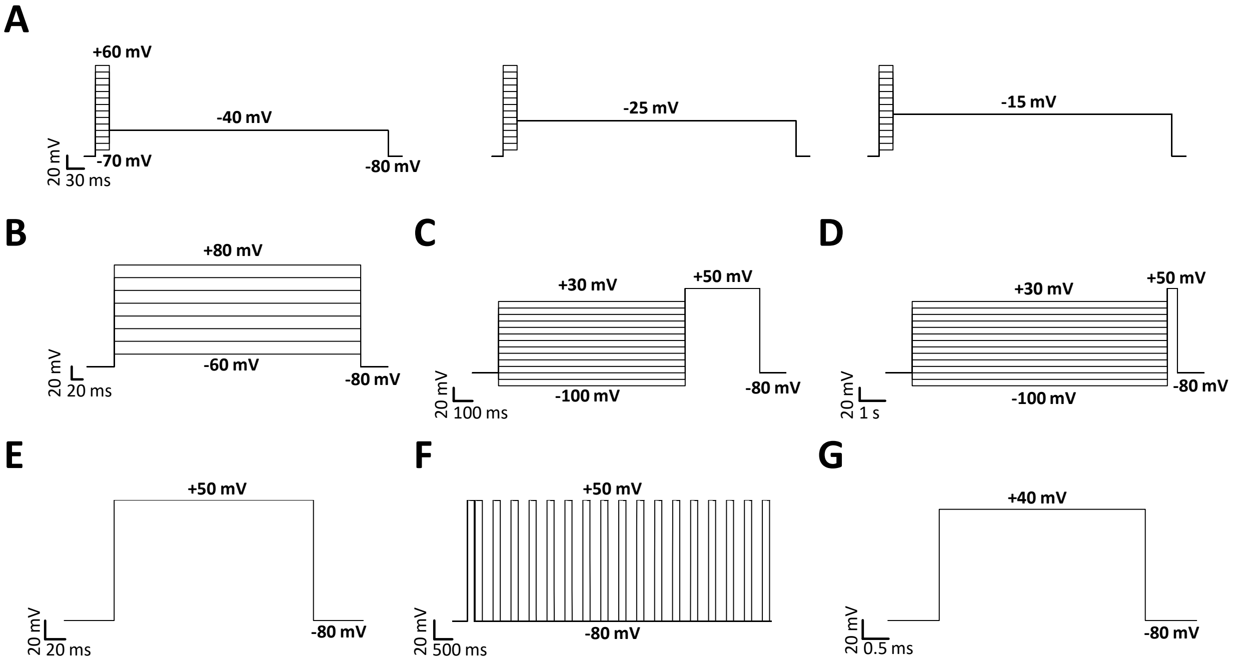

Supplementary Figure 5. Voltage protocols used for voltage clamp recordings to analyse **(A)** the conductance-voltage relationship (GV) **(B)** the current voltage relationship (IV) **(C)** **(D)** the kinetics of inactivation **(E)** the current amplitude **(F)** the kinetics of the recovery from inactivation and **(G)** the frequency dependence of inactivation accumulation.

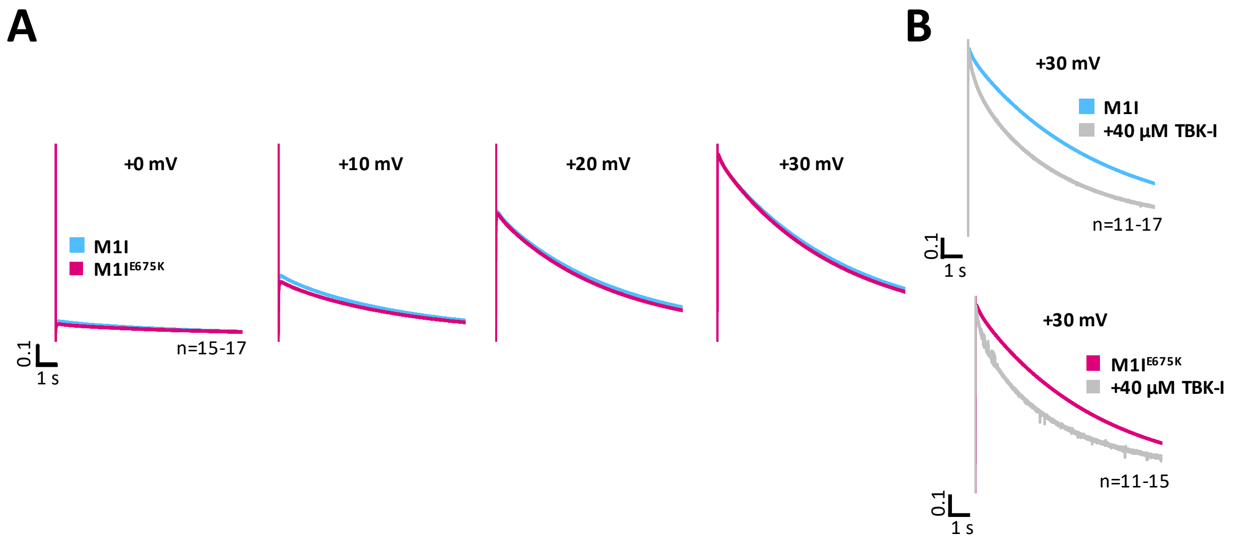

Supplementary Figure 6. Inactivation kinetics and TBK1 modulation of Kv3.3^{M1I} and the Kv3.3$^{M1I/E675K}$ variant. **(A)** Averaged current traces to illustrate the voltage-dependence of activation and the inactivation kinetics at different potentials for the Kv3.3^{M1I} and the Kv3.3$^{M1I/E675K}$ variant. The voltage protocol is illustrated in Supplementary Figure 6B). **(B)** Averaged current traces to illustrate the inactivation kinetics of the Kv3.3^{M1I} and the Kv3.3$^{M1I+E675K}$ variant without or after pre-treatment with 40 µM of the TBK1 inhibitor MRT67307. The time constants of inactivation of the Kv3.3^{M1I} variant before and after TBK1 inhibitor treatment were: $\tau1$ = 6.79 s, $\tau2$ = 0.04 s, A1/(A1+A2) = 0.96; $\tau1$ = 4.48 s, $\tau2$ = 0.15 s, A1/(A1+A2) = 0.83. The time constants of inactivation of the Kv3.3$^{M1I/E675K}$ variant before and after TBK1 inhibitor treatment were: $\tau1$ = 6.51 s, $\tau2$ = 0.03 s, A1/(A1+A2) = 0.97; $\tau1$ = 4.21 s, $\tau2$ = 0.14 s, A1/(A1+A2) = 0.83. The number of replicates is indicated within the graph.

1.2 Supplementary Tables

Supplementary Table 1. Previously described mutations in the *KCNC3* gene associated with gain- or loss-of-function effects on the Kv3.3 channel found in SCA13 patients.

Mutation	Effects on channel function	Source
**p.D129N**	left-shift in $V_{1/2}$	Duarri et al., 2015
**p.R420H**	complete loss-of-function<br>dominant negative effect on Kv3.3 WT	Waters et al., 2006<br>Duarri et al., 2015
**p.R423H**	complete loss-of-function<br>dominant negative effect on Kv3.3 WT	Figueroa et al., 2010<br>Figueroa et al., 2011
**p.T428I**	complete loss-of-function<br>dominant negative effect on Kv3.3 WT<br>slowed activation kinetics of Kv3.3/Kv3.3^{T428I}	Nemeth et al., 2013<br>Parolin Schnekenberg et al., 2015
**p.F448L**	left-shift in the $V_{1/2}$<br>slowed deactivation kinetics	Waters et al., 2006
**p.V535M**	left-shift in $V_{1/2}$	Duarri et al., 2015
**p.S591G**	right-shift in $V_{1/2}$<br>reduced current density	Duarri et al., 2015
**p.G592R**	slowed inactivation kinetics<br>disrupted actin cytoskeleton interactions<br>increase in cell death	Zhang et al., 2016<br>Zhang et al., 2021b

Duarri, A., Nibbeling, E.A., Fokkens, M.R., Meijer, M., Boerrigter, M., Verschuuren-Bemelmans, C.C., et al. (2015). Functional analysis helps to define *KCNC3* mutational spectrum in Dutch ataxia cases. *PLoS One* 10, e0116599. doi: 10.1371/journal.pone.0116599

Figueroa, K.P., Minassian, N.A., Stevanin, G., Waters, M., Garibyan, V., Forlani, S., et al. (2010). *KCNC3*: phenotype, mutations, channel biophysics-a study of 260 familial ataxia patients. *Hum Mutat* 31, 191-196. doi: 10.1002/humu.21165

Figueroa, K.P., Waters, M.F., Garibyan, V., Bird, T.D., Gomez, C.M., Ranum, L.P., et al. (2011). Frequency of *KCNC3* DNA variants as causes of spinocerebellar ataxia 13 (SCA13). *PLoS One* 6, e17811. doi: 10.1371/journal.pone.0017811

Nemeth, A.H., Kwasniewska, A.C., Lise, S., Parolin Schnekenberg, R., Becker, E.B., Bera, K.D., et al. (2013). Next generation sequencing for molecular diagnosis of neurological disorders using ataxias as a model. *Brain* 136, 3106-3118. doi: 10.1093/brain/awt236

Parolin Schnekenberg, R., Perkins, E.M., Miller, J.W., Davies, W.I., D'Adamo, M.C., Pessia, M., et al. (2015). De novo point mutations in patients diagnosed with ataxic cerebral palsy. *Brain* 138, 1817-1832. doi: 10.1093/brain/awv117

Waters, M.F., Minassian, N.A., Stevanin, G., Figueroa, K.P., Bannister, J.P., Nolte, D., et al. (2006). Mutations in voltage-gated potassium channel *KCNC3* cause degenerative and developmental central nervous system phenotypes. *Nat Genet* 38, 447-451. doi: 10.1038/ng1758

Zhang, Y., Varela, L., Szigeti-Buck, K., Williams, A., Stoiljkovic, M., Sestan-Pesa, M., et al. (2021). Cerebellar Kv3.3 potassium channels activate TANK-binding kinase 1 to regulate trafficking of the cell survival protein Hax-1. *Nat Commun* 12, 1731. doi: 10.1038/s41467-021-22003-8

Zhang, Y., Zhang, X.F., Fleming, M.R., Amiri, A., El-Hassar, L., Surguchev, A.A., et al. (2016). Kv3.3 Channels Bind Hax-1 and Arp2/3 to Assemble a Stable Local Actin Network that Regulates Channel Gating. *Cell* 165, 434-448. doi: 10.1016/j.cell.2016.02.009

Supplementary Table 2. Genes sequenced in the clinical exome approach.

AAAS	*AHSG*	*ARSA*	*BAG3*	*CATSPER2*	*CIITA*	*COQ5*	*DIABLO*
AARS1	*AIFM1*	*ARSB*	*BBS1*	*CAV3*	*CISD2*	*COQ6*	*DIAPH1*
AARS2	*AIP*	*ARSL*	*BBS12*	*CBL*	*CLCN2*	*COQ8A*	*DIAPH3*
AASS	*AIPL1*	*ARVCF*	*BBS2*	*CC2D2A*	*CLCN7*	*COX10*	*DISP1*
ABCA12	*AK2*	*ASAH1*	*BBS4*	*CCBE1*	*CLCNKA*	*CP*	*DKC1*
ABCA2	*AKAP9*	*ASCL1*	*BBS5*	*CCDC103*	*CLCNKB*	*CPLANE1*	*DLAT*
ABCA4	*AKT1*	*ASL*	*BBS9*	*CCDC39*	*CLN5*	*CPS1*	*DLD*
ABCA7	*ALAD*	*ASNS*	*BCKDHA*	*CCDC40*	*CLRN1*	*CRB1*	*DLL1*
ABCB6	*ALDH18A1*	*ASPM*	*BCKDHB*	*CCDC50*	*CNGA1*	*CREBBP*	*DMD*
ABCC1	*ALDH5A1*	*ASS1*	*BCL11A*	*CCDC88C*	*CNGB1*	*CRIPTO*	*DMP1*
ABCC6	*ALG11*	*ATCAY*	*BCOR*	*CCND1*	*CNNM2*	*CRPPA*	*DNAAF1*
ABCC8	*ALG3*	*ATG7*	*BCR*	*CCR1*	*CNOT3*	*CRTAP*	*DNAAF11*
ABCC9	*ALG6*	*ATL1*	*BDNF*	*CD109*	*CNTNAP2*	*CRYAB*	*DNAAF2*
ABCD1	*ALG8*	*ATM*	*BEST1*	*CD151*	*COCH*	*CRYM*	*DNAAF3*
ABHD12	*ALG9*	*ATN1*	*BICRA*	*CDC42BPB*	*COG1*	*CSF1R*	*DNAAF4*
ACADM	*ALMS1*	*ATP10A*	*BMP1*	*CDC6*	*COG4*	*CSRP3*	*DNAAF5*
ACADS	*ALOX12B*	*ATP13A2*	*BMP2*	*CDH1*	*COG5*	*CTC1*	*DNAH11*
ACAT1	*ALOXE3*	*ATP1A2*	*BMP4*	*CDH23*	*COG7*	*CTDP1*	*DNAH5*
ACBD5	*ALS2*	*ATP1A3*	*BRAF*	*CDHR1*	*COL11A1*	*CTH*	*DNAH9*
ACOX1	*ALX1*	*ATP2B2*	*BRCA1*	*CDK5RAP2*	*COL11A2*	*CTSA*	*DNAI2*
ACP5	*ALX3*	*ATP6AP2*	*BRCA2*	*CDON*	*COL18A1*	*CTSK*	*DNAL1*
ACSL4	*AMACR*	*ATP6V0A1*	*BRIP1*	*CDT1*	*COL1A1*	*CUL4B*	*DNASE1L3*
ACTA1	*ANK1*	*ATP6V0A2*	*BSCL2*	*CEACAM16*	*COL1A2*	*CXCR4*	*DNM1*
ACTB	*ANK2*	*ATP6V0A4*	*BSND*	*CEP290*	*COL25A1*	*CYP7B1*	*DNMT1*
ACTC1	*ANKH*	*ATP6V1B1*	*BTD*	*CEP41*	*COL2A1*	*DARS2*	*DNMT3A*
ACTG1	*ANKRD11*	*ATP7A*	*BTK*	*CEP57*	*COL3A1*	*DBH*	*DNMT3B*
ACTN2	*ANO10*	*ATP8A2*	*BTRC*	*CERKL*	*COL4A1*	*DBT*	*DOCK6*
ACVR1	*ANOS1*	*ATP8B1*	*C19orf12*	*CFAP418*	*COL4A3*	*DCAF17*	*DOCK7*
ADAR	*ANTXR1*	*ATPAF2*	*C4A*	*CFTR*	*COL4A4*	*DCC*	*DPAGT1*
ADGRG1	*AP1S1*	*ATRX*	*CA2*	*CHAT*	*COL4A5*	*DCDC2*	*DPM1*
ADGRV1	*AP4E1*	*ATXN10*	*CA8*	*CHD2*	*COL4A6*	*DDB2*	*DSG2*
ADK	*AP4M1*	*ATXN2*	*CACNA1A*	*CHD3*	*COL6A1*	*DDR2*	*DSP*
ADSL	*AP5Z1*	*ATXN3*	*CACNA1C*	*CHD7*	*COL6A2*	*DDX11*	*DSPP*
AFF2	*APC*	*ATXN8OS*	*CACNA1D*	*CHD8*	*COL6A3*	*DEAF1*	*DUOX2*
AFF3	*APOB*	*AUH*	*CACNA1G*	*CHN1*	*COL9A1*	*DES*	*DUOXA2*
AFG3L2	*APP*	*AUTS2*	*CACNA2D1*	*CHRNA7*	*COL9A2*	*DGUOK*	*DYM*
AGA	*APTX*	*B3GLCT*	*CAMTA1*	*CHRNG*	*COL9A3*	*DHCR24*	*DYRK1A*
AGO2	*ARID1A*	*B4GALNT1*	*CASK*	*CHST14*	*COLEC11*	*DHCR7*	*EBP*
AGRN	*ARID1B*	*B9D1*	*CASQ2*	*CHSY1*	*COMT*	*DHODH*	*EDN1*
AHI1	*ARL13B*	*B9D2*	*CAT*	*CIB2*	*COQ2*	*DHX16*	*EDNRA*

EDNRB	*FANCL*	*G6PC3*	*GNE*	*HK1*	*ITGA2B*	*KRAS*	*MCOLN1*
EFTUD2	*FANCM*	*GAA*	*GNPTAB*	*HLA-B*	*ITGB3*	*KRT5*	*MECP2*
EHMT1	*FAS*	*GABRA5*	*GNRH1*	*HLA-DPB1*	*ITM2B*	*KYNU*	*MED13L*
ELAC2	*FBN1*	*GABRB3*	*GNRHR*	*HLA-DQA1*	*ITPR1*	*L2HGDH*	*MED25*
ELN	*FBXW7*	*GABRD*	*GNS*	*HLA-DQB1*	*IYD*	*LAMA1*	*MEFV*
ELOVL4	*FGF3*	*GABRG2*	*GOSR2*	*HLA-DRB1*	*JAG1*	*LAMA4*	*MEN1*
ELP1	*FGF9*	*GAD1*	*GP1BA*	*HLCS*	*JMJD1C*	*LAMB1*	*MERTK*
ENPP1	*FGFR2*	*GALC*	*GPC3*	*HMGCL*	*JPH2*	*LARGE1*	*MET*
EP300	*FGFR3*	*GALE*	*GPC4*	*HNF1B*	*JUP*	*LARS2*	*MFSD2A*
EPAS1	*FGFRL1*	*GALK1*	*GPI*	*HOMER2*	*KANSL1*	*LCA5*	*MGP*
EPM2A	*FH*	*GALNS*	*GPSM2*	*HOXA1*	*KARS1*	*LDB3*	*MIA3*
ERAP1	*FHL1*	*GALNT2*	*GRHL2*	*HS6ST1*	*KCNA1*	*LETM1*	*MID1*
ERBB2	*FHL2*	*GAS2L2*	*GRIA3*	*HSD17B4*	*KCNA5*	*LHX3*	*MINPP1*
ERBB3	*FIG4*	*GATA2*	*GRIK2*	*HSPD1*	*KCNAB2*	*LHX4*	*MKS1*
ERCC1	*FIP1L1*	*GATA3*	*GRIP1*	*HSPG2*	*KCNC3*	*LIG3*	*MLC1*
ERCC2	*FKBP10*	*GATA4*	*GRM1*	*HTRA1*	*KCND3*	*LITAF*	*MLXIPL*
ERCC3	*FKBP14*	*GATAD1*	*GRM7*	*HTT*	*KCNE1*	*LMBRD1*	*MMACHC*
ERCC4	*FKBP6*	*GBA1*	*GRN*	*HUWE1*	*KCNE5*	*LMNA*	*MME*
ERCC5	*FKRP*	*GBE1*	*GRXCR1*	*HYDIN*	*KCNH2*	*LMNB1*	*MPDZ*
ERCC6	*FKTN*	*GCGR*	*GSDME*	*IDH3B*	*KCNJ11*	*LMNB2*	*MPV17*
ERCC8	*FLCN*	*GCK*	*GSN*	*IDS*	*KCNJ13*	*LMX1B*	*MPZ*
ERLIN2	*FLNA*	*GCLC*	*GSS*	*IDUA*	*KCNJ3*	*LORICRIN*	*MRE11*
ESPN	*FLNB*	*GFAP*	*GSTM3*	*IFIH1*	*KCNJ5*	*LOXHD1*	*MRPS22*
ESRRB	*FLVCR1*	*GFER*	*GTF2IRD1*	*IFNG*	*KCNMA1*	*LRP2*	*MSH4*
ETHE1	*FMR1*	*GJA5*	*GTF2IRD2*	*IFNGR1*	*KCNN3*	*LRP4*	*MSRB3*
EXOSC3	*FOXH1*	*GJA8*	*GUCA1B*	*IFRD1*	*KCNQ1*	*LRP5*	*MSX1*
EXT1	*FOXI1*	*GJB1*	*GUCY2D*	*IFT140*	*KCNQ2*	*LRPPRC*	*MTFMT*
EYA1	*FOXP1*	*GJB3*	*GUSB*	*IL10*	*KCNQ4*	*LTBP1*	*MTHFD1*
EYA4	*FOXP2*	*GLA*	*HACE1*	*IL23R*	*KDM5C*	*LYST*	*MTHFR*
EYS	*FOXRED1*	*GLB1*	*HARS2*	*ILDR1*	*KDM6A*	*MADD*	*MTPAP*
EZH2	*FRAS1*	*GLE1*	*HAX1*	*IMPDH1*	*KIF1A*	*MAF*	*MTRR*
FA2H	*FREM2*	*GLI2*	*HDAC4*	*IMPG2*	*KIF1B*	*MAK*	*MTTP*
FAM161A	*FRG1*	*GLI3*	*HDAC8*	*INF2*	*KIF21A*	*MAN1B1*	*MYBPC3*
FAM20C	*FRMPD4*	*GLIS3*	*HEPACAM*	*INPP5E*	*KIF5A*	*MAN2B1*	*MYH11*
FANCB	*FSCN2*	*GLRA1*	*HEXB*	*INS*	*KIF7*	*MANBA*	*MYH14*
FANCC	*FSHR*	*GLRB*	*HFE*	*INVS*	*KISS1R*	*MAP2K1*	*MYH3*
FANCD2	*FTO*	*GLRX5*	*HGD*	*IQCB1*	*KIT*	*MARVELD2*	*MYH6*
FANCF	*FUCA1*	*GLYCTK*	*HGF*	*IQSEC2*	*KITLG*	*MASP1*	*MYH7*
FANCG	*FUS*	*GNAQ*	*HGSNAT*	*IRF6*	*KMT2C*	*MBD5*	*MYH9*
FANCI	*FXN*	*GNAS*	*HIBCH*	*ITGA2*	*KMT2D*	*MCM3AP*	*MYL2*

MYO15A	*NIPA1*	*PALB2*	*PIK3R1*	*PRPF31*	*RNASEH2B*	*SDCCAG8*	*SLC26A2*
MYO3A	*NIPAL4*	*PANK2*	*PIK3R5*	*PRPF8*	*RNASET2*	*SDHA*	*SLC26A5*
MYO5A	*NIPBL*	*PAX2*	*PLA2G6*	*PRPH2*	*RNF168*	*SDHAF1*	*SLC26A9*
MYO6	*NKX2-5*	*PAX3*	*PLAGL1*	*PRRT2*	*ROBO1*	*SDHAF2*	*SLC29A3*
MYO7A	*NLRP12*	*PAX7*	*PLCB4*	*PSAT1*	*ROBO3*	*SDHB*	*SLC35A1*
MYPN	*NLRP3*	*PCDH15*	*PLEC*	*PSEN1*	*ROGDI*	*SDHD*	*SLC35C1*
NAA10	*NME8*	*PCNT*	*PLEKHG4*	*PSEN2*	*ROM1*	*SEC23B*	*SLC39A4*
NAGA	*NODAL*	*PDE4D*	*PLP1*	*PTCH1*	*ROR2*	*SEMA3A*	*SLC4A1*
NAGLU	*NOP10*	*PDE6A*	*PLXND1*	*PTEN*	*RORA*	*SEMA3E*	*SLC4A11*
NAGS	*NOP56*	*PDE6B*	*PML*	*PTH1R*	*RP1*	*SEMA4A*	*SLC52A3*
NARS2	*NOS1AP*	*PDE6G*	*PMM2*	*PTPN22*	*RP1L1*	*SERPINB6*	*SLC5A5*
NCF1	*NOTCH2*	*PDGFB*	*PMP22*	*PTPRQ*	*RP9*	*SETBP1*	*SLC5A7*
NDE1	*NOTCH3*	*PDGFRA*	*PNPLA1*	*PTS*	*RPGR*	*SETD2*	*SLC6A19*
NDRG1	*NPC1*	*PDHA1*	*PNPLA2*	*RAB18*	*RPGRIP1*	*SETX*	*SLC6A5*
NDST1	*NPHP1*	*PDHB*	*PNPLA6*	*RAB3GAP1*	*RPGRIP1L*	*SF3B4*	*SLC6A8*
NDUFA11	*NPHP3*	*PDHX*	*POGZ*	*RAB3GAP2*	*RPL10*	*SGCD*	*SLC9A6*
NDUFA4	*NPHP4*	*PDSS1*	*POLD1*	*RAC1*	*RPL11*	*SGSH*	*SLX4*
NDUFA6	*NPM1*	*PDZD7*	*POLG*	*RAD21*	*RPS26*	*SH2B1*	*SMARCA2*
NDUFA9	*NR2E3*	*PEPD*	*POLR1D*	*RAD50*	*RRM2B*	*SH3TC2*	*SMARCA4*
NDUFAF1	*NR4A2*	*PEX1*	*POLR3A*	*RAD51*	*RSPH4A*	*SHANK3*	*SMARCB1*
NDUFAF2	*NR5A1*	*PEX10*	*POLR3B*	*RAD51C*	*RYR1*	*SHH*	*SMC1A*
NDUFAF4	*NSD1*	*PEX14*	*POMGNT1*	*RAF1*	*RYR2*	*SHOC2*	*SMC3*
NDUFAF6	*NSDHL*	*PEX16*	*POMT1*	*RAI1*	*SACS*	*SIGMAR1*	*SMPD1*
NDUFS1	*NSUN2*	*PEX19*	*POMT2*	*RALGAPA1*	*SALL1*	*SIL1*	*SMS*
NDUFS2	*NTRK2*	*PEX2*	*POR*	*RBM10*	*SALL4*	*SIM1*	*SNAI2*
NDUFS4	*NUBPL*	*PEX3*	*POU3F4*	*RBM20*	*SARDH*	*SIX1*	*SNAP29*
NDUFS6	*NUP155*	*PEX5*	*PPP2R2B*	*RBM8A*	*SATB2*	*SIX3*	*SNRNP200*
NDUFS7	*NUP62*	*PEX6*	*PPT1*	*RDH12*	*SBDS*	*SIX5*	*SNTA1*
NDUFS8	*OAT*	*PHEX*	*PQBP1*	*RDH5*	*SBF2*	*SIX6*	*SNX10*
NDUFV1	*OCA2*	*PHF6*	*PRCD*	*RDX*	*SCN10A*	*SLC11A1*	*SORL1*
NDUFV2	*OFD1*	*PHGDH*	*PRF1*	*RECQL4*	*SCN1A*	*SLC12A3*	*SOS1*
NEB	*OPA3*	*PHOX2A*	*PRICKLE1*	*RET*	*SCN1B*	*SLC12A6*	*SOX6*
NECTIN1	*ORC4*	*PHYH*	*PRKAR1A*	*REV3L*	*SCN2A*	*SLC16A2*	*SOX9*
NEDD4L	*ORC6*	*PIEZO2*	*PRKCG*	*RFC2*	*SCN2B*	*SLC17A5*	*SP7*
NEFL	*OTC*	*PIGA*	*PRKDC*	*RFT1*	*SCN3B*	*SLC17A8*	*SPATA7*
NEK1	*OTOA*	*PIGL*	*PRODH*	*RFX5*	*SCN4A*	*SLC19A2*	*SPG11*
NEXN	*OTOF*	*PIGN*	*PROKR2*	*RFXAP*	*SCN4B*	*SLC19A3*	*SPG21*
NF1	*OTX2*	*PIGO*	*PROM1*	*RGR*	*SCN5A*	*SLC1A2*	*SPG7*
NF2	*OXR1*	*PIK3CA*	*PROP1*	*RIC1*	*SCN8A*	*SLC1A3*	*SPRED1*
NHLRC1	*PAFAH1B1*	*PIK3CD*	*PRPF3*	*RIPK4*	*SCN9A*	*SLC25A12*	*SPTA1*

SPTB	*SYNGAP1*	*TCTN1*	*TMEM67*	*TPRN*	*TULP1*	*VLDLR*	*XRCC1*
SPTBN2	*SYT14*	*TCTN2*	*TMIE*	*TRAF3IP1*	*TXNRD2*	*VPS13B*	*XYLT2*
SPTLC2	*SYT2*	*TDO2*	*TMPRSS3*	*TRDN*	*TYMP*	*VRK1*	*YAP1*
SQSTM1	*TAFAZZIN*	*TENM4*	*TNC*	*TREX1*	*TYMS*	*VSX1*	*YARS1*
SRCAP	*TAT*	*TERT*	*TNFRSF11A*	*TRIO*	*TYR*	*WASHC4*	*YWHAE*
SREBF1	*TBCD*	*TG*	*TNFRSF11B*	*TRIOBP*	*UBA1*	*WASHC5*	*YY1*
SRPX2	*TBCE*	*TGFB1*	*TNFRSF1A*	*TRPM4*	*UBAC2*	*WDPCP*	*ZEB2*
STAT3	*TBK1*	*TGIF1*	*TNFSF11*	*TRPS1*	*UBE3C*	*WDR11*	*ZFP57*
STAT5B	*TBL1XR1*	*TGM1*	*TNNI3*	*TRPV3*	*UBR1*	*WDR19*	*ZFYVE26*
STIL	*TBP*	*TGM6*	*TNNT2*	*TRPV4*	*UFD1*	*WDR4*	*ZIC2*
STRC	*TBX1*	*TH*	*TOMM40*	*TRRAP*	*UNC13D*	*WDR81*	*ZMPSTE24*
STX1A	*TBX15*	*THPO*	*TOPORS*	*TSC2*	*UNC80*	*WFS1*	*ZNF469*
STXBP2	*TBX22*	*TLR4*	*TOR1A*	*TSHZ1*	*UROC1*	*WHRN*	*ZNF526*
SUCLA2	*TBX4*	*TMC1*	*TP63*	*TSPAN12*	*USH1C*	*WNT10B*	*ZNF592*
SUFU	*TBX5*	*TMCO1*	*TPK1*	*TTBK2*	*USH2A*	*WNT4*	
SULT2B1	*TCAP*	*TMEM126A*	*TPM1*	*TTC19*	*USP7*	*WRAP53*	
SUMF1	*TCF4*	*TMEM127*	*TPM2*	*TTC8*	*USP9X*	*WT1*	
SUOX	*TCIRG1*	*TMEM216*	*TPM3*	*TTN*	*VCL*	*WWOX*	
SUPT16H	*TCN2*	*TMEM237*	*TPO*	*TTPA*	*VCP*	*XPC*	
SYNE1	*TCOF1*	*TMEM43*	*TPP1*	*TUBB3*	*VDR*	*XPNPEP3*	

Supplementary Table 3. Activation kinetics of wild-type Kv3.3 and Kv3.3^{E675K}. Kinetics were measured with an IV protocol described in the Methods section and Supplementary Figure 5B.

	**Kv3.3**	**Kv3.3^{E675K}**	**Significance**
**+20 mV**	4.27 ± 0.42 ms (n=10)	4.48 ± 0.26 ms (n=15)	n.s.
**+40 mV**	1.72 ± 0.18 ms (n=10)	1.75 ± 0.11 ms (n=15)	n.s.
**+60 mV**	0.83 ± 0.13 ms (n=10)	0.94 ± 0.07 ms (n=15)	n.s.
**+80 mV**	0.66 ± 0.12 ms (n=9)	0.94 ± 0.14 ms (n=15)	n.s.

n.s., not significant. The number of experiments is indicated in parentheses.

Supplementary Table 4. Deactivation kinetics of wild-type Kv3.3 and Kv3.3^{E675K}. Deactivation kinetics of the tail currents were analysed, following a pre-step to +40 mV for 25 ms and a test pulse to -50 mV, -40 mV, -30 mV, -20 mV or -10 mV, respectively, for 400 ms to analyse the kinetics of the tail currents.

	**Kv3.3**	**Kv3.3^{E675K}**	**Significance**
**-10 mV**	2.74 ± 0.05 ms (n=8)	2.58 ± 0.20 ms (n=9)	n.s.
**-20 mV**	1.88 ± 0.09 ms (n=16)	1.79 ± 0.12 ms (n=15)	n.s.
**-30 mV**	1.20 ± 0.04 ms (n=16)	1.14 ± 0.08 ms (n=15)	n.s.
**-40 mV**	0.81 ± 0.07 ms (n=15)	0.78 ± 0.07 ms (n=13)	n.s.
**-50 mV**	0.53 ± 0.06 ms (n=9)	0.60 ± 0.11 ms (n=6)	n.s.

n.s., not significant. The number of experiments is indicated in parentheses.

Supplementary Table 5. Comparison of frequency- and $[K^+]_{ex}$-dependent accumulation of inactivation (relative currents) between wild-type Kv3.3 and Kv3.3^{E675K}.

	1 mM $[K^+]_{ex}$		8 mM $[K^+]_{ex}$		98 mM $[K^+]_{ex}$	
	**Kv3.3**	**E675K**	**Kv3.3**	**E675K**	**Kv3.3**	**E675K**
**1 Hz**	0.95± 0.01	0.92 ± 0.01*	0.97 ± 0.01	0.95 ± 0.00*	1.00 ± 0.01	1.00 ± 0.01 n.s.
**10 Hz**	0.64 ± 0.04	0.49 ± 0.02**	0.79 ± 0.03	0.66 ± 0.02***	0.96 ± 0.1	0.94 ± 0.01 n.s.
**50 Hz**	0.25 ± 0.03	0.16 ± 0.01*	0.40 ± 0.04	0.25 ± 0.01**	0.83 ± 0.04	0.70 ± 0.03*
**100 Hz**	0.14 ± 0.02	0.1 ± 0.01 n.s.	0.21 ± 0.03	0.12 ± 0.01**	0.55 ± 0.05	0.41 ± 0.03*

n.s., not significant. *, $p < 0.05$; **, $p < 0.01$; ***, $p < 0.001$. For the number of experiments (n) see Figure 8.
